# Cytotoxic CD4^+^ T-follicular cells may mediate killing against lymphoma cells

**DOI:** 10.3389/fimmu.2025.1657046

**Published:** 2025-09-15

**Authors:** Yin Xiao, Sigrun S. Haeusl, Gaurav Jethva, Johannes Weber, Andreas Rosenwald, Friederike Berberich-Siebelt

**Affiliations:** ^1^ Institute of Pathology, Julius-Maximilians-University Würzburg, Würzburg, Germany; ^2^ Comprehensive Cancer Centre Mainfranken, Julius-Maximilians-University of Würzburg, Würzburg, Germany

**Keywords:** follicular lymphoma, cytotoxic CD4^+^ T cells, diffuse large B-cell lymphoma, GZMK, GZMB, NKG-7, T-follicular helper cells

## Abstract

Recently, we have identified CD4^+^PD-1^+^CXCR5^+^ T-follicular helper (T_FH_) cells with a distinct cytotoxic phenotype and named them “killer T_FH_ (T_F_ **_K_**)” cells. In this study, we aim to elucidate their presence and functional relevance in two different lymphoma subtypes, follicular lymphoma (FL) and diffuse large B-cell lymphoma (DLBCL). Flow cytometric analysis of tonsillar versus FL-cell suspensions revealed a heightened number of GZMK^+^NKG7/TIA-1^+^ T_F_ **_K_** cells in the latter, accompanied by a significant increase in T-regulatory and T-follicular regulatory (T_FR_) cells. In contrast, DLBCL exhibited a decrease in T_FH_ and T_FR_ cell numbers, while concurrently demonstrating heightened frequencies of GZMK^+^TIA-1^+^ and especially GZMB^+^TIA-1^+^ T_F_ **_K_** cells within the T_FH_ population. Analysis of single-cell RNA sequencing data confirmed an origin-specific phenotype of T_F_ **_K_** cells. Immunofluorescence staining of biopsy specimens detected CD4^+^BCL-6^+^TIA-1^+^ T_F_ **_K_** cells within follicles and germinal centers (GC) in reactive lymph nodes and within their atypical counterparts in malignant lymph nodes. Their propensity to migrate into atypical GCs was more pronounced in higher grade FLs. Furthermore, the release of cytotoxic cargo by degranulation could be induced by stimulation of CD4^+^ cells in cultures of FL and DLBCL suspensions. In line, the direct cytotoxic capacity of T_F_ **_K_** cells against lymphoma cells was demonstrated by killing assays with isolated cells, underscoring their potential as a prospective therapeutic target in lymphoma control.

## Introduction

Cytotoxic CD4^+^ T cells have been identified during periods of chronic inflammation and are understood to be reprogrammed T-helper cells that retain their MHCII restriction and manifest a terminally differentiated phenotype. These cells contribute to host defense through their direct cytolytic capacity to kill infected cells (1–5). Cytotoxic CD4^+^ T cells occur in supercentenarians making a signature of healthy aging (6), but can also correlate with active progressive disease as exemplified by CNS-resident CD4^+^EOMES^+^GZMB^+^NR4A2^+^ T cells in secondary progressive multiple sclerosis (7).

Lymphopenic mice revealed that CD4^+^ T cells in those expand with a cytotoxic phenotype and are sufficient to eradicate melanoma (8, 9). Concurrently, CD4^+^ T cells are regarded as anti-tumor effector cell (10). A preponderance of reports document an activated T-helper 1 (T_H_1) phenotype of cytotoxic CD4^+^ T cells, including tumor microenvironment (TME)-associated ones, while alternate polarization patterns have been observed (11). These cells require antigen for their proliferation and respond to elevated IL-2 levels, which can occur in the absence of Treg cells. Direct tumor-killing capability has been reported (12–14).

We recently discovered a small subset of CD3^+^CD4^+^CD45RA^–^CXCR5^+^ T cells with a cytotoxic phenotype in peripheral blood as well as in tonsils of rather healthy individuals (15). As the chemokine receptor CXCR5 facilitates homing of lymphocytes to B-cell follicles building germinal centers (GC), the majority of CXCR5^+^ cells are specialized CD4^+^ T lymphocytes providing cognate help to GC-B cells, hence the term T-follicular helper (T_FH_) cells (16, 17). T_FH_ cells are further characterized by high expression of other surface molecules, such as ICOS or PD-1, the transcription factor BCL-6, the cytokines IL-21 and IL-4, and – in humans – the chemokine CXCL13.

This newly identified cytotoxic T_FH_ subtype was distinguished by high expression of NKG7 (Natural Killer Cell Granule Protein 7), granzymes, perforin, and CCL5. It could be triggered to degranulate its cytotoxic cargo, ultimately leading to its designation as a “killer T_FH_” (T_F_ **_K_**) cell. Notably, while T_F_ **_K_** cells express CXCR5 and BCL6, they co-express EOMES and BLIMP-1 (encoded by *PRDM1*), which may be crucial for their cytotoxic function (18, 19). BLIMP-1 and the detected LITAF have been shown to engage in a reciprocal negative loop with BCL-6 (20, 21). However, the co-expression of BLIMP-1 and BCL6 in T_F_ **_K_** cells reflects the situation in CXCR5^+^FOXP3^+^BLIMP-1^+^ T-follicular regulatory (T_FR_) cells, in which the level of BCL6 is indeed less than in T_FH_ cells (22). Additionally, T_F_ **_K_** cells express markers of type 1 terminal differentiation, including CXCR3, CX3CR1 and KLRG1. In line with CXCR3 expression, T_F_ **_K_** cells mostly remained in an extrafollicular position in the tonsils. Yet they shared TCR specificities with the majority of the other CD3^+^CD4^+^CD45RA^–^CXCR5^+^ subclusters, including classical GC-T_FH_ cells and this was despite their predominant oligoclonal profile due to a constrained TCR diversity (15).

Several types of B-cell lymphomas express GC-B-cell signature genes. Follicular lymphoma (FL) is an indolent lymphoma that can transform into an aggressive subtype (transformed FL, most frequently to DLBCL) (23). In most cases FL is marked by a t(14;18) translocation, leading to BCL-2 overexpression (24). During affinity maturation within the GC, where only a few GC-B cells can receive survival signals from T_FH_ cells, BCL-2-overexpressing B cells have an advantage and avoid apoptosis. These pre-cancerous cells acquire activation-induced deaminase (AID)-induced mutations within the GC. Cellular interactions in the GC-like TME of an FL resemble benign immune reactions (25). In lymph node (LN)-localized FL, follicular dendritic cells, fibroblastic reticular cells and T_FH_ cells are the major components of the TME, further accompanied by Tregs, various CD4^+^ and CD8^+^ T-cell types, whose presence and frequency define subtypes of FL (26, 27).

DLBCL is classified based on gene expression patterns that define the cell of origin, i.e. two biologically and clinically distinct subtypes, namely GCB-DLBCL and ABC-DLBCL, representing the B-cell differentiation stages of GC (GCB) and post-GC (ABC) (28, 29). Several driver mutations and an overall severe genetic heterogeneity further characterize the subgroups (30). Like FL, GCB-DLBCL is transcriptionally reminiscent of light zone GC-B cells, but in all morphological variants any follicular structures have vanished (31).

Here, we show that lymphoma samples contain cytotoxic CD4^+^ T cells, which were predominantly NKG7/TIA-1^+^GZMK^+^ T_FH_-like in FL and TIA-1^+^GZMK^+^ and/or GZMB^+^ within the CXCR5^+^PD-1^+^ and CXCR5^–^PD-1^+^ subpopulations of DLBCL. In non-malignant, but reactive LNs and malignant FL these T_F_ **_K_** cells were predominantly located in the follicles and GCs, while the degree of malignancy further supports their appearance in GCs. Tregs were enriched in number but rarely present in close contact with T_F_ **_K_** cells. Single-cell RNA-seq datasets confirmed the abundant presence of cytotoxic CD4^+^ T cells in FL and DLBCL with origin-specific transcriptomes. Degranulation assays restricted to CD4-MHCII interactions indicated the ability for degranulation and cytotoxicity, while isolated and stimulated T_F_ **_K_** cells could induce apoptosis in FL and DLBCL B cells, highlighting their potential role in anti-lymphoma immunity.

## Materials and methods

### Patient samples for cell suspensions and histology

Malignant LNs with FL and DLBCL had been collected in comparison to tonsils from patients undergoing tonsillectomy (**Supplementary Table S1**) at the Medical Faculty of the Julius-Maximilians-University Würzburg. After isolation of mononuclear cells (MNC) using cell sieves, cells were frozen and stored in liquid nitrogen at the Institute of Pathology.

Human tissue samples of FL, DLBCL, and reactive LNs (**Supplementary Table S1**), together with their clinical data, were available at the Institute of Pathology. All relevant histologic examinations were completed. Ethical approval was obtained for this study, i.e., the data were used according to the ethical guidelines of the Medical Faculty and approved by the Institutional Ethics Committee of the Julius-Maximilians-University (149/23 and 136/21). Informed consent was obtained after a written explanation of the nature and possible consequences of the studies (136/21), while in 149/23 it is residual diagnostic material that has been approved for research by the Ethics Committee. Therefore, our study adheres to the Declaration of Helsinki.

### Flow cytometric analysis of human MNC from LN and non-malignant tonsils

A total of 5 × 10^6^ cells were used for flow cytometry analysis. Viable cells were first identified using the Zombie NIR™ Fixable Viability Kit or Zombie Green™ Fixable Viability Kit (both from BioLegend). Following viability staining, cells were incubated with Human TrueStain FcX™ (BioLegend) for 10 minutes to block Fc receptors. Surface staining was then performed at room temperature (RT) for 15 minutes using the following fluorophore-conjugated antibodies (**Supplementary Table S2**): BV510- or PerCP or FITC-conjugated CD4 (clone OKT4, BioLegend), APC-Cy7- or PE-Cy7-conjugated PD-1 (clone NAT105, BioLegend), PerCP-conjugated CD19 (clone, HIB19), Pacific Blue-conjugated CXCR5 (clone I252D4, BioLegend), PerCP-conjugated CD107a (clone H4A3, BioLegend), PE-Cy7-conjugated ICOS (clone C398.4A, BioLegend), and PE-conjugated TIA-1 (clone 2G9A10F5, Beckman Coulter). Following surface staining, cells were fixed and permeabilized using the FOXP3/Transcription Factor Staining Buffer Set (Invitrogen) according to the manufacturer’s instructions. Intracellular staining was subsequently performed using FITC-conjugated FOXP3 (clone 206D, BioLegend), APC-conjugated Granzyme K (clone GM26E7, BioLegend), and BV510-conjugated Granzyme B (clone GB11, BD Horizon™). Data were acquired on a FACSCanto II (BD Biosciences) flow cytometer and analyzed with FlowJo^®^ v10.8.1 (Treestar Inc., Ashland, OR, USA).

### Immunofluorescence histology staining and analysis

Consecutive formalin-fixed paraffin-embedded (FFPE) sections were used to localize T_F_ **_K_** cells. The procedure of staining and image analysis has been described in detail (15). Briefly, the tissue sections were processed through deparaffinization followed by heat-induced antigen retrieval in citrate buffer (pH 6.0). After blocking with Dako protein block (DAKO, S3022) for 1 h at room temperature, sections were incubated with primary antibodies (**Supplementary Table S3**) for 1 h, washed three times with TBST, and then stained with appropriate secondary antibodies (**Supplementary Table S3**) alongside DAPI for 1 h at room temperature. Following final washes, slides were mounted with Fluoromount-G (ThermoFisher).

A Zeiss LSM780 confocal microscope was used for image acquisition. The 20x/0.8 objective and the required channels were selected. The smart-set-up of the microscope was set to “best signal”, while the detection range for the four channels was set to minimize signal spillover from the different channels. Prior to image acquisition, the GC was located using DAPI staining of the nuclei. The gain of the laser had to be adjusted according to the tissue, as LNs and FLs stained differently under the microscope.

The raw confocal images were then processed using Fiji (Fiji is just ImageJ. The steps of deconvolution, segmentation and cell counting were achieved as described (15). In addition, background correction could now be included in the macro before deconvolution. Cell subtype counting was achieved by applying a second macro, which used the Colocalization Finder. The actual overlap of the two images was transferred as regions of interest (ROIs) to the ROI manager, where the pixel size of each single overlap ROI was measured. If the ROI was larger than 200 pixels or smaller than 20 pixels, the ROI was discarded and deleted. Subsequently, the results were transferred into an Excel spreadsheet containing the sample number, cell subtype as well as ROI number and size. This plugin was used once more to indicate possible locations of T_F**K**_ cells. T_F**K**_ cell candidates were confirmed by creating intensity profiles as described (15). The process of creating and saving intensity profiles could now be automated using a Fiji macro. The measurement of follicles and GC sizes was automated as well, after manually specifying and creating ROIs for all follicles and GCs according to CD19 and BCL6 staining. The results were saved automatically in an Excel spreadsheet. The allocation of T_F**K**_ cells to follicles and GCs was done according to the aforementioned ROIs of follicles and GCs. T_F**K**_ numbers and all other cell numbers were collected in an Excel spreadsheet where sample averages and percentages were calculated.

For the cell-cell contact analysis of T_F_ **_K_** cells with FOXP3^+^ Treg cells, CD4^+^ T cells were first identified by a Fiji plugin and afterwards checked by CellProfiler for at least 25% overlay with signal in the FOXP3 channel. The outlines of the Treg cells were superimposed on the merged image and manually checked for cell-cell contact with T_F_ **_K_** cells.

### scRNAseq

We used publicly available scRNAseq and TCRseq datasets to query gene expression as well as TCR usage in T_F_ **_K_** cells of FL and DLBCL compared to tonsils. Samples and sequencing procedure can be found in (32).

### scRNA-seq bioinformatics

All scRNA-seq data (32) were processed and analyzed using Seurat (v5.2.1). The FindAllMarkers function was applied to identify differentially expressed genes and to annotate clusters; however, gene set enrichment analysis (GSEA), KEGG pathway analysis, RITAN, and enrichR were not utilized in this study.

For TCR reconstruction and paired TCR clonotype calling, Cell Ranger (version 3.0.2) was used for variable diversity joining sequence assembly. TCR analysis was performed using scRepertoire (v2.2.1), where instead of random 1000, all sequenced cells were selected and single TCRα/TCRβ genes were retained for analysis.

### *In vitro* stimulation of adenoid cultures

*In vitro* stimulation with staphylococcal enterotoxin B (SEB; Sigma S4881-1MG) was performed as previously described (15). Cells from tonsils, FL and DLBCL were plated in 96 well U-plate (Greiner bio-one), 2*10^5 cells/well were stimulated with 1 μg/ml of SEB for 4 days. For assessment of degranulation capacity, PerCP-conjugated CD107a (H4A3, Biolegend) antibody, with or without monensin (Biolegend) and brefeldin A (Biolegend), was added during the last 3 hours of stimulation. Samples were acquired on a FACS Canto II (BD Biosciences) flow cytometer.

### T_F**K**_ cell isolation and killing assay

The killing assay was optimized based on the methodology described (33). Fluorescence-activated cell sorting (FACS) was used to isolate T_F**K**_ cells (CD4^+^CD25^–^CXCR5^+^PD1^+^TIA-1^+^), T_FH_ cells (CD4^+^CD25^–^CXCR5^+^PD1^+^TIA-1^–^), and B cells (CD19^+^) from FL and DLBCL samples. Afterwards, B cells were incubated with 10 µg/mL anti-CD3 (clone: OKT3, BioLegend) for 1 hour at 37 °C to serve as target cells. Subsequently, anti-CD3 mAb-coated B cells were co-cultured with either T_FH_ or T_F**K**_ cells at a 1:1 ratio in a 96 well U-plate (Greiner bio-one) for 14 hours. After incubation, apoptotic target cells were quantified by flow cytometry following staining with the Zombie NIR™ Fixable Viability Kit (Biolegend) and APC-conjugated Annexin V (BioLegend) according to the manufacturer’s instructions.

### Statistical analysis

All flow cytometric data are shown as mean ± SD and represent combined data from at least three independent experiments. The results were analyzed with Prism software (GraphPad) using Wilcoxon signed-rank test, Kruskal-Wallis One-way ANOVA. * *p* < 0.05, **p < 0.005, *** *p* < 0.001, ****p < 0.0001.

All statistical analyses and their respective graphs presented for IF staining were conducted created with GraphPad Prism by using T-test or Mann-Whitney test. * *p* < 0.05, **p < 0.005, *** *p* < 0.001, ****p < 0.0001.

## Results

### Germinal center-derived lymphoma harbor cytotoxic CD4^+^CXCR5^+^ T cells

Recently, we detected a subcluster of CD3^+^CD4^+^CD45RA^–^CXCR5^+^ T_FH_ cells with a cytotoxic phenotype in peripheral blood and tonsils of rather healthy individuals (15). This observation prompted us to investigate whether GC-derived lymphoma harbor such cytotoxic T_FH_ – hereafter referred to as killer T_FH_ or T_F**K**_ cells. To address this, we performed flow cytometry analysis on samples from 13 FL and 9 DLBCL patients, comparing them with tonsil specimens from non-cancer patients (**Supplementary Figures S1A, B**).

**Figure 1:**
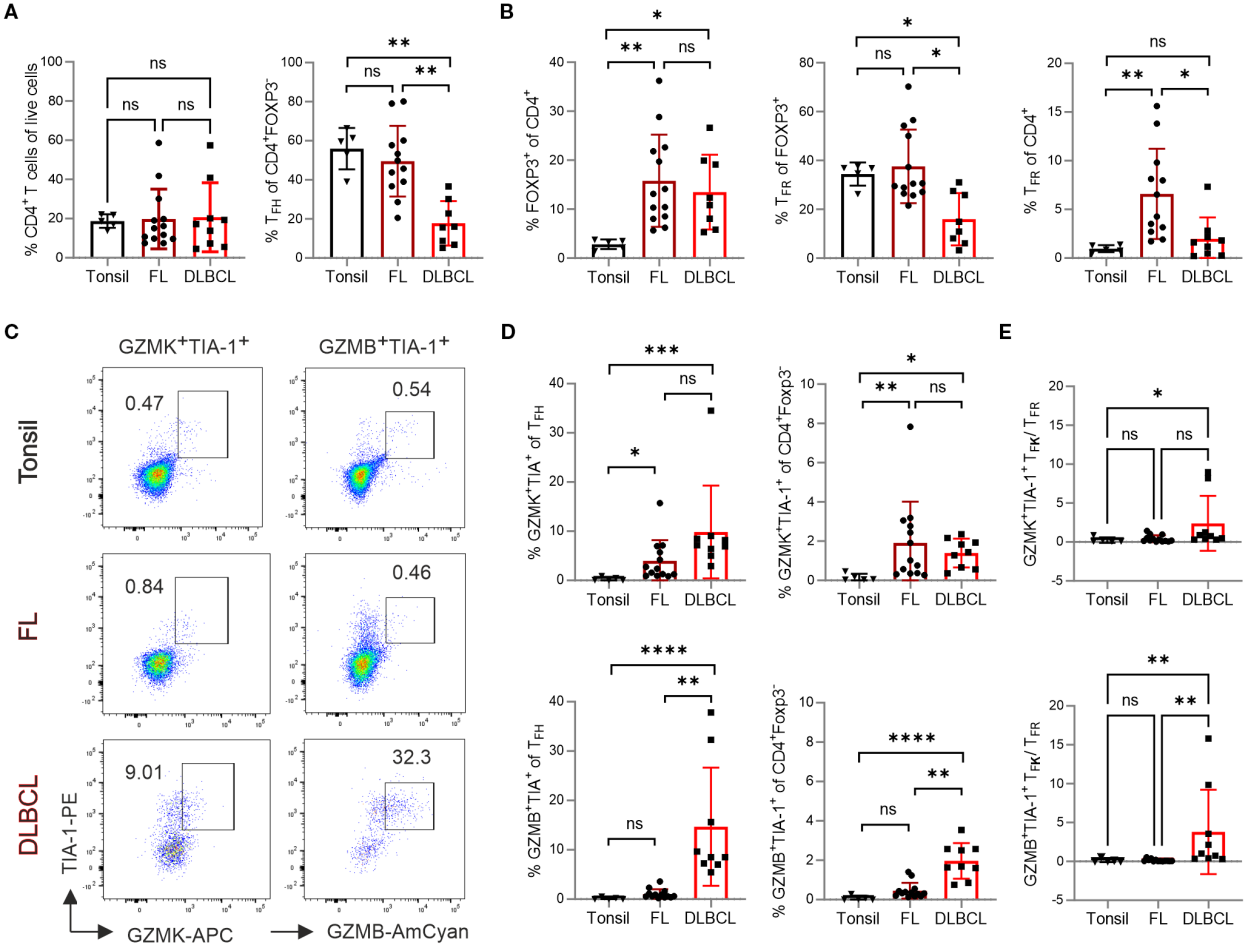
T_F**K**_ cells are more frequent in FL and DLBCL patients. Flow cytometric analysis of different T-cell subpopulations in samples from tonsil (n=5), FL (n=13), and DLBCL (n=9). **(A)** Frequency of total CD4^+^ T cells and T_FH_ cells. **(B)** Frequency of FOXP3^+^ regulatory T (Treg) cells and T_FR_ cells. **(C)** Representative dot plots showing GZMK^+^TIA-1^+^ and GZMB^+^TIA-1^+^ T_FH_ cells. **(D)** Frequency of GZMK^+^TIA-1^+^ and GZMB^+^TIA-1^+^ cells within T_FH_ cells and CD4^+^Foxp3^-^ T cells, respectively. **(E)** The ratio of GZMK^+^TIA-1^+^ or GZMB^+^TIA-1^+^ T_F**K**_ cells to T_FR_ cells. Kruskal-Wallis One-way ANOVA, ns, non-significant, *p < 0.05, **p < 0.005, ***p < 0.001, ****p < 0.0001.

While CD4^+^ T cells were equally abundant in tonsils, FL and DLBCL, only FL samples exhibited a comparable proportion of CXCR5^+^PD-1^+^ T_FH_ within the CD4^+^FOXP3^–^ conventional T cells (Tconv) relative to tonsils (**Figure 1A**). Both lymphomas contained significantly more FOXP3^+^ Tregs, which was reflected in more CD4^+^FOXP3^+^CXCR5^+^PD-1^+^ T_FR_ cells among CD4^+^ T cells, but not among Tregs for FL (**Figure 1B**). In contrast to FL, DLBCL samples exhibited a significant reduction in both T_FH_ and T_FR_ cells. Furthermore, DLBCL had significantly fewer T_FR_ among CD4^+^FOXP3^+^ Tregs compared to tonsil.

T_F**K**_ cells are characterized by high expression of NKG7 and granzymes, particularly the tryptases GZMK and GZMA, with lower level of GZMB (15). Given that human NKG7 can be recognized by the anti-TIA-1 antibody (clone 2G9A10F5) - which exhibits well-documented cross-reactivity with both TIA-1 and NKG7 through recognition of a conserved pentameric epitope (GYETQ in TIA-1 and GYETL in NKG7 (34)). We therefore quantified the number of GZMK^+^TIA-1^+^ or GZMB^+^TIA-1^+^ T_F**K**_ cells within the CD4^+^FOXP3^–^CXCR5^+^PD-1^+^ T_FH_ population (**Figure 1C**). Compared to tonsils, both FL and DLBCL showed a higher frequency of GZMK^+^TIA-1^+^ T_F**K**_ cells within T_FH_ or total CD4^+^ Tconv cells (**Figure 1D**). In contrast, the frequency of GZMB^+^TIA-1^+^ T_F**K**_ cells was similarly low in tonsils and FL. Notably, DLBCL samples exhibited a relative enrichment of GZMB^+^TIA-1^+^ T_F**K**_ cells within both the CXCR5^+^PD-1^+^ T_FH_ and the total CD4^+^ Tconv population. Furthermore, the positive correlation between T_F**K**_ cell abundance (both GZMK^+^TIA-1^+^ and GZMB^+^TIA-1^+^) and malignancy grade was confirmed by analyzing FL samples stratified by transformation stage (**Supplementary Figures S2A, B**). Given that T_FR_ cells have been implicated in suppressing GZMK^+^ cytotoxic T_FH_ cells in ectopic lymphoid follicles of patients with IgG4-related disease (35), we assessed the ratio of GZMK^+^TIA-1^+^ or GZMB^+^TIA-1^+^ T_F**K**_ cells to T_FR_ cells. Notably both T_F**K**_ cell subsets outnumbered T_FR_ cells in DLBCL samples, while this imbalance was not observed in FL (**Figure 1E**).

In summary, compared to tonsillar samples, FL exhibited an increased frequency of T_F**K**_ cells, predominately of the GZMK^+^TIA-1^+^ phenotype, accompanied by significantly elevated Tregs and T_FR_ cells. In contrast, DLBCL showed reduced numbers of T_FH_ and T_FR_ cells, but displayed enrichment of both GZMK^+^TIA-1^+^ and GZMB^+^TIA-1^+^ T_F**K**_ cells as well as Tregs.

### CD4^+^FOXP3^–^PD-1^+^ T-cell cytotoxicity decreases with CXCR5 expression in FL but not in DLBCL

**Figure 2:**
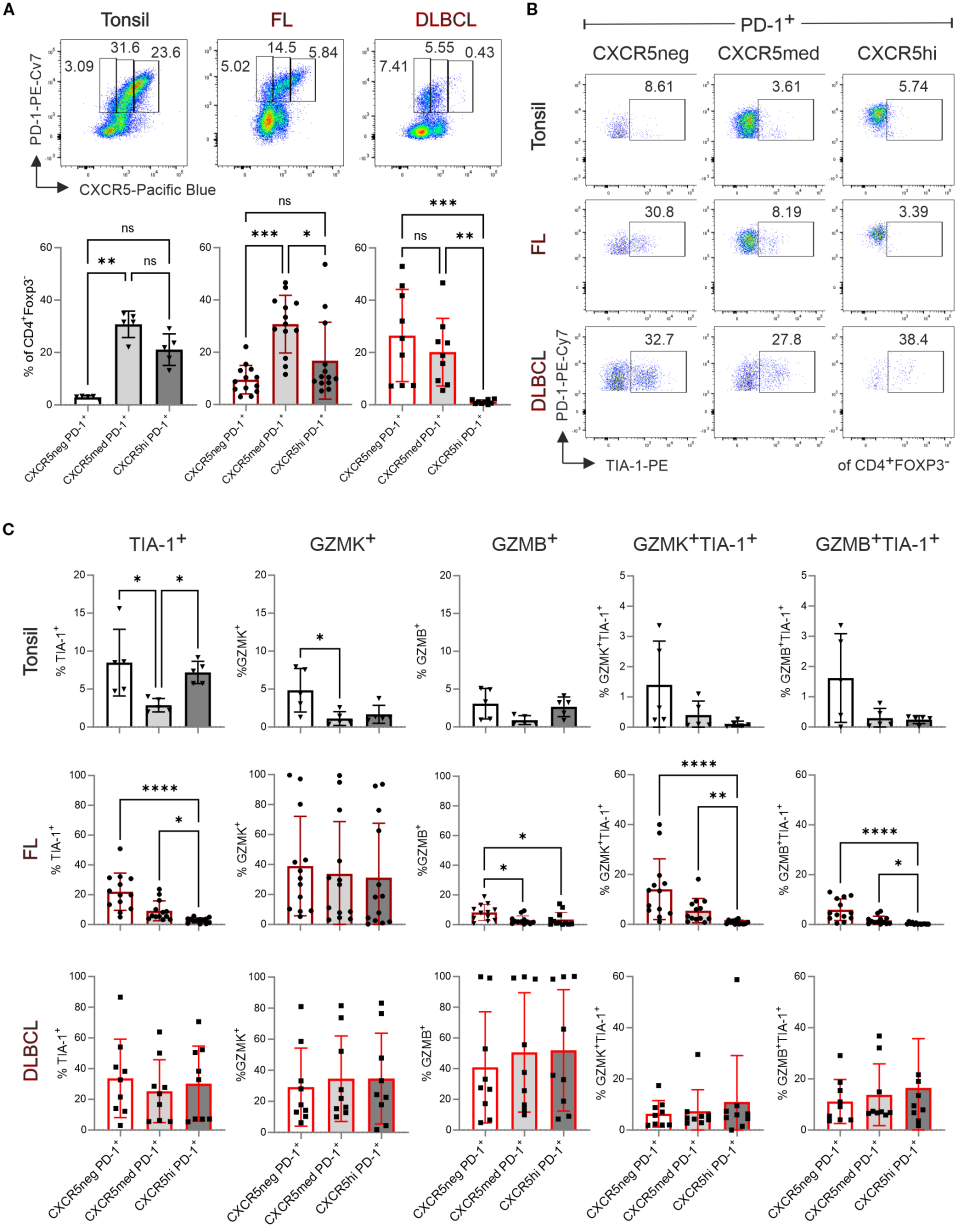
T_F**K**_ cell frequency decreases with increasing CXCR5 expression in tonsils and FL but remains unchanged in DLBCL. Flow cytometric analysis of T-cell subpopulations in tonsil (n=5), FL (n=13), and DLBCL (n=9). **(A)** Representative dot plots (top panel) and frequency (bottom panel) of CXCR5^–^PD-1^+^, CXCR5^med^PD-1^+^, and CXCR5^hi^PD-1^+^ cells in tonsil, FL, and DLBCL samples. **(B)** Representative dot plots showing TIA-1 expression in CXCR5^–^, CXCR5^med^, and CXCR5^hi^ populations. **(C)** Frequency of TIA-1^+^, GZMK^+^, GZMB^+^, GZMK^+^TIA-1^+^, and GZMB^+^TIA-1^+^ cells within CXCR5^–^PD-1^+^, CXCR5^med^PD-1^+^, and CXCR5^hi^PD-1^+^ population. Kruskal-Wallis One-way ANOVA, ns, non-significant, *p < 0.05, **p < 0.005, ***p < 0.001, ****p < 0.0001.

Next, we aimed to determine whether tonsils, FL and DLBCL also contain cytotoxic non-T_FH_, defined as CD4^+^PD-1^+^CXCR5^–^TIA-1^+^ cells. Simultaneously we assessed CXCR5 expression level on the GZMK^+^TIA-1^+^ and GZMB^+^TIA-1^+^ T_F**K**_ cells. For classification, we distinguished between PD-1^+^CXCR5^med^ T_FH_ and PD-1^+^CXCR5^hi^ GC-T_FH_ cells (16). In both tonsils and FL, most CD4^+^FOXP3^–^PD-1^+^ T cells express CXCR5 (**Figure 2A**). However, FL-derived CD4^+^FOXP3^–^PD-1^+^CXCR5^+^ cells exhibited intermediate CXCR5 expression level. In DLBCL, where T_FH_ cells were less abundant (**Figure 1A**), the proportions of CD4^+^PD-1^+^CXCR5^–^ and CD4^+^PD-1^+^CXCR5^+^ cells were roughly equal, while GC-T_FH_ cells were underrepresented (**Figure 2A**). Interestingly, in tonsils, TIA-1^+^CD4^+^FOXP3^–^PD-1^+^ T cells were similarly frequent among CXCR5^–^ and CXCR5^hi^ subsets, though granzyme expression tended to decrease with increasing CXCR5 levels (**Figures 2B, C**; **Supplementary Figures S3A-C**). In FL, NKG7/TIA-1^+^ cells were predominantly non-T_FH_ cells, less frequent in CXCR5^med^ T_FH_ cells, and rare in CXCR5^hi^ T_FH_ cells. Most TIA-1^+^ cells co-expressed granzymes, particularly GZMK in FL, while GZMK levels remained comparable across CXCR5^–^, CXCR5^med^, and CXCR5^hi^CD4^+^PD-1^+^ T cells. In DLBCL, no significant differences in cytotoxic phenotypes were observed among the analyzed T-cell subsets. However, CD4^+^FOXP3^–^PD-1^+^ T cells displayed generally high expression of TIA-1, GZMK, and GZMB, independent of CXCR5 expression level.

### T_F**K**_ cells localize in a higher likelihood to the atypical GCs in FL than to GCs in reactive LNs

**Figure 3:**
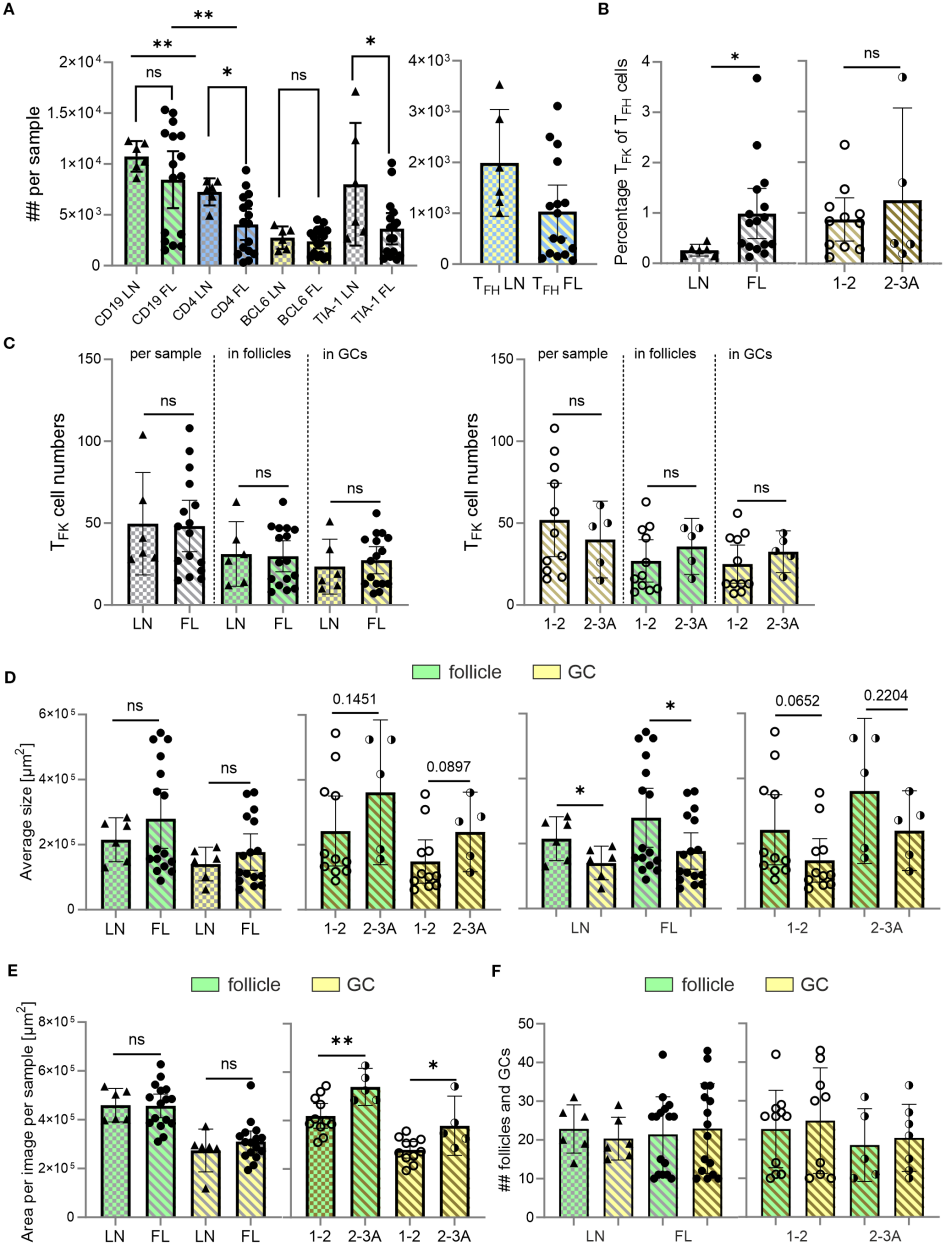
Despite no differences in T_F**K**_ cell numbers, the percentage of T_F**K**_ within T_FH_ cells is enhanced in malignant versus non-malignant LNs. CD19 and follicles (green), CD4 (blue), BCL-6 and GCs (yellow), TIA-1 (cyan), FL grade 1-2 (light golden stripes), FL 2-3 (dark golden stripes). **(A)** Absolute cell numbers per sample of CD19^+^ B cells, CD4^+^ T cells, BCL6^+^ follicular cells, and TIA-1^+^ cells in non-malignant LNs and FL. **(B)** Percentage of T_F_ **_K_** of T_FH_ cells for LN, FL grade 1–2 and FL grade 2-3A. **(C)** T_F_ **_K_** numbers between LN and low and intermediate graded FL in total, follicles or GCs. **(D)** Average follicle and GC size compared between FL and LN, as well as compared with each other within the same tissue origin. **(E)** Area covered by follicles and GCs in non-malignant LNs, and FL grade 1–2 or grade 2-3. **(F)** Absolute number of follicles and GCs in FL and LN. **(A-F)** Mann Whitney T-test, ns, non-significant, *p < 0.05, **p < 0.005.

Being CXCR5^+^, T_F**K**_ cells could home to GCs. However, T_F**K**_ cells in non-malignant tonsils are mainly localized extrafollicularly (15). To investigate their distribution in malignant tissues, we performed IF staining on FL samples, which maintain defined (though atypical) follicular structures with atypical GCs and partially preserved mantle zones, comparing them to reactive LNs as non-malignant controls. Using antibodies against CD19, CD4, TIA-1 and BCL6, we identified CD19^+^BCL6^+^ GC-B cells, CD4^+^BCL6^+^TIA-1^–^ T_FH_ and CD4^+^BCL6^+^TIA-1^+^ T_F**K**_ cells. In both tissues we found single T_F**K**_ cells, e.g. localized outside the follicles (**Supplementary Figures S4**, **S5**). Computer-assisted quantification uncovered comparable numbers of CD19^+^ B cells and total BCL6^+^ cells between FL and reactive LNs. However, FL samples contained fewer CD4^+^ T cells, fewer TIA-1^+^ cells, and a trend towards reduced T_FH_ cell numbers (**Figure 3A**). Strikingly, the percentage of CD4^+^TIA-1^+^BCL6^+^ T_F**K**_ cells within the CD4^+^BCL6^+^ T_FH_ population was significantly higher in FL compared to reactive LNs (**Figure 3B**), although their cell numbers—whether total per sample, follicular, or GC-localized—did not differ significantly (**Figure 3C**). Morphometric analysis showed that atypical follicles and GCs in FL tended to be larger than their benign ones in reactive LNs. A similar trend was observed when comparing low-grade (grade 1–2) and intermediate-grade (grade 2–3A) FLs (**Figure 3D**). This size difference became statistically significant when quantified per image per sample (**Figure 3E**). In contrast, neither the number of follicles/GCs nor their frequency correlated with disease severity (**Figure 3F**).

**Figure 4:**
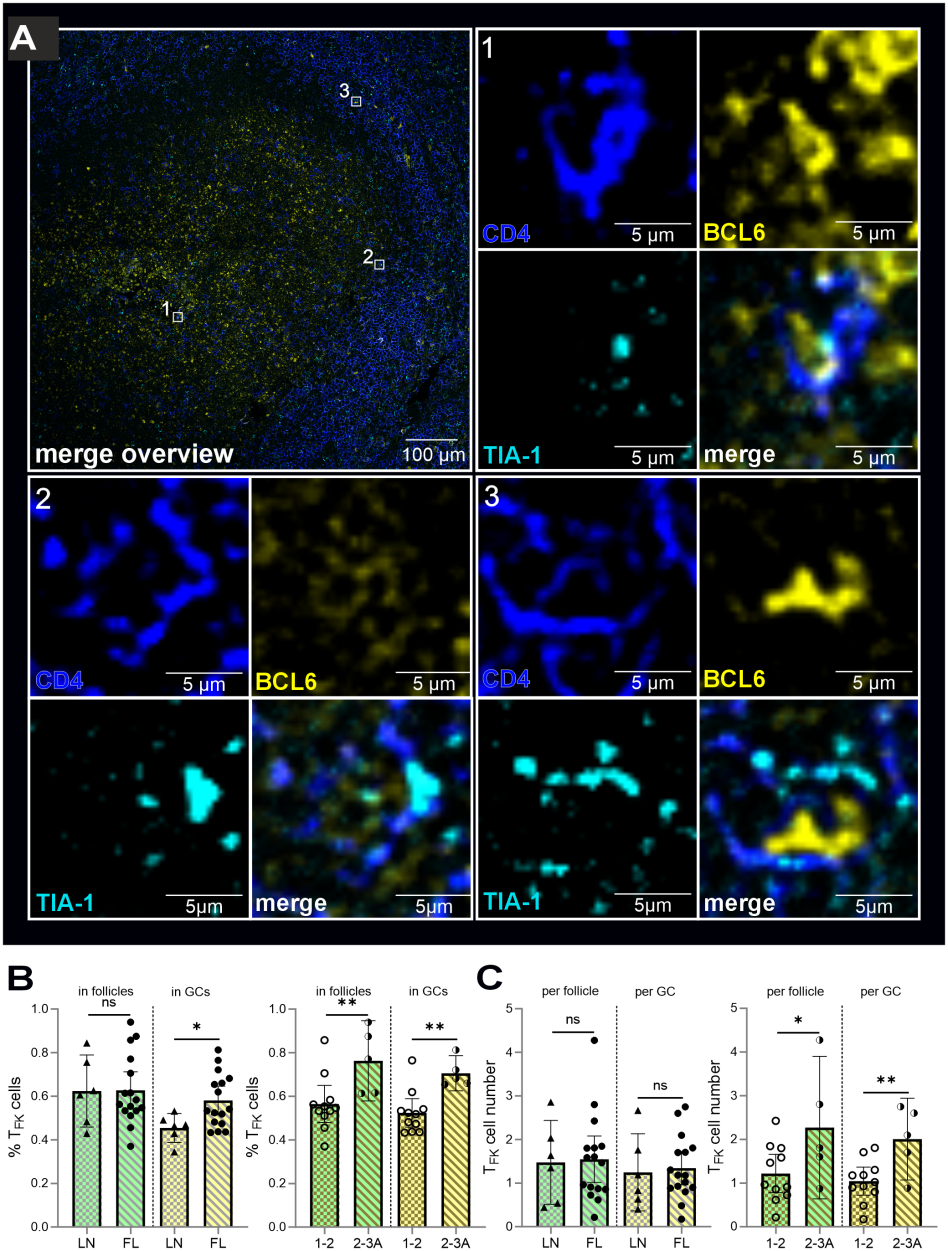
T_F**K**_ cells are more likely to be located in the atypical GCs of FL than in the benign GCs of non-malignant LN. CD4 (blue), BCL6 and GCs (yellow), TIA-1 (blue), follicles (green), FL grade 1-2 (light golden stripes), FL 2-3 (dark golden stripes). **(A)** T_F_ **_K_** cells in “GCs” of FL. Each cell is indicated in the overview. All three separate channels are shown for each cell as well as the merged images. **(B)** Percentage of T_F_ **_K_** cells of all detected T_F**K**_ cells within follicles and GCs of non-malignant versus FL-LNs, FL grade 1–2 and FL grade 2-3A. **(C)** Differences in absolute T_F_ **_K_** cell numbers in non-malignant versus FL-LN as well as in low (1-2) and intermediate (2-3A) graded FL for atypical follicle and “GC”. **(A-C)** Mann Whitney T-test, ns, non-signigicant, *p < 0.05, **p < 0.005).

Thus, immunofluorescence analysis unequivocally identified CD4^+^TIA-1^+^BCL6^+^ T_F**K**_ within the atypical follicles and GCs of FL tissues (**Figure 4A**). Interestingly, their relative abundance in GCs/GC-like structures showed a significant stepwise increase with disease progression—first from reactive LN to FL overall, and then with disease severity (**Figure 4B**). This led to higher T_F**K**_ cell numbers in the atypical follicles and especially in “GCs” of grade 2-3A FLs compared to grade 1–2 FLs (**Figure 4C**). In summary, while CD4^+^TIA-1^+^BCL6^+^ T_F**K**_ cells are present in both reactive and malignant LNs, their follicular and GC localization is markedly more pronounced in FL than in mildly inflamed tonsils (15). Importantly, we observed that T_F**K**_ cell frequencies among T_FH_ cells and their GC homing propensity were highest in more advanced FL grades, suggesting a potential association between T_F**K**_ cell accumulation and disease malignancy.

### Single cell RNA sequencing CD4^+^ T cell confirmed the presence of T_F**K**_ cells in FL and DLBCL

To further characterize the NKG7^+^/TIA-1^+^ CD4^+^FOXP3^–^PD-1^+^CXCR5^+^ in FL and DLBCL, we could rely on publicly available scRNAseq data (32). Spasevska et al. had been interested to define the phenotype of intratumoral Tregs in FL and DLBCL and sequenced CD4^+^ T cells from three FL, DLBCL and non-malignant tonsils each. Besides three Treg clusters, they defined 10 clusters of CD4^+^ Tconv cells of which two resembled T_FH_ cells (*PDCD1*, *CXCR5*, *IL21*, and *TOX2*) and one displayed upregulated RNA for *GZMK*, *NKG7*, *CST7*, *GZMA*, *GZMB, and PRF1* (32).

**Figure 5:**
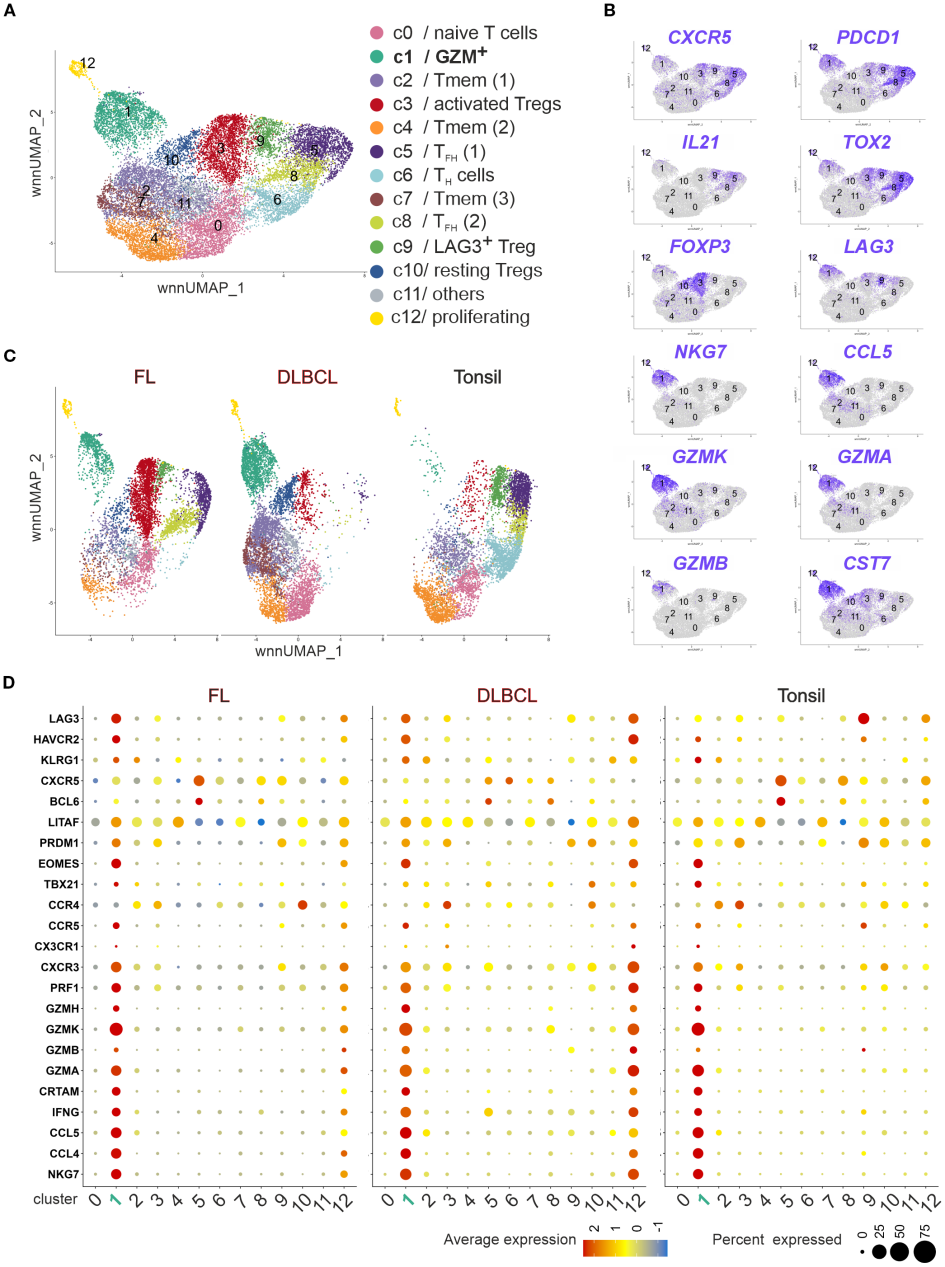
Tonsils, FL, and DLBCL harbor cytotoxic CD4^+^ T cells with a T_F**K**_ transcriptome. Publicly available scRNAseq data of CD4^+^ T cells of three tonsils, three FL and three DLBCL lymphoma (32) were reanalyzed regarding their cytotoxic phenotype. **(A)** wnnUMAP of pooled CD4^+^ T cells (n = 18,771 cells, roughly 2,000/donor). Each dot corresponds to a single cell, color-indexed according to its cluster affiliation (c0-c12, cluster color code and annotation corresponding to (32). **(B)** Feature plots of T_FH_, Treg and T_F_ **_K_** gene markers on simplified wnnUMAPs. **(C)** wnnUMAPs separated by origin. **(D)** Bubble plots to project T_F_ **_K_** gene markers to each cluster in FL, DLBCL or tonsils. The size of a dot corresponds to the percentage of cells expressing the # feature in each cluster. The color represents the average expression level.

We reanalyzed the data using Seurat version (v5.2.1) and projected them in a *weighted-nearest neighbor uniform manifold approximation and projection* (wnnUMAP) defining the same clusters (c0 – c12; **Figure 5A**). Simplified UMAPs showed higher *CXCR5*, *PDCD1*, *IL21* and *TOX2* RNA expression in T_FH_ c5 and c8 as well as *FOXP3* in c3 and c10 or *LAG3* in c9/FOXP3^–^LAG3^+^ Tregs (32) (**Figure 5B**). c1 [designated as GZM^+^ (32)] exhibited almost exclusive RNA expression of *NKG7*, *CCL5*, *GZMK*, *GZMA*, *GZMB*, and *CST7* (encoding cystatin-F). However, cells in c1 also shared the expression of *CXCR5*, *PDCD1*, *TOX2*, or *LAG3* with T_FH_ cells, activated FOXP3^+^ and FOXP3^–^LAG3^+^ Tregs, albeit *CXCR5* to a lesser extent than in T_FH_ cells. Still, this classified c1 as a subtype of follicular cells, before named T_F**K**_ cells by us (15). As a minimum it can be stated that c1 contained T_F**K**_ and that c3 contained T_FR_ cells as well as that c9/LAG3^+^ Tregs, expressing CXCR5, PD-1, TOX2, and IL-21, resembled T_FH_-like cells.

In agreement with our flow cytometric data, the three sequenced DLBCL samples enclosed almost no non-cytotoxic T_FH_ cells (c5 and c8 or c9; **Figure 5C**). However, while only a limited number of cells from GZM^+^ c1 could be identified in tonsils, FL exhibited approximately 10% and DLBCL 20% cytotoxic CD4^+^ T cells (**Figure 5C**). Plotting the signature genes of T_F**K**_ cells to all clusters in FL, DLBCL, and tonsils revealed the presence of them in tissues of each origin (**Figure 5D**). Interestingly, in FL and more pronounced in DLBCL the small cluster of proliferating CD4^+^ T cells (c12) acquired a similar cytotoxic phenotype. Taken together, despite lower *CXCR5* and *BCL6* levels than in T_FH_ cells, tonsils, FL and DLBCL harbored cytotoxic CD4^+^ T cells with a T_F**K**_ phenotype, confirming the T_FH_-like phenotype of cytotoxic CD4^+^ T cells found in FL (26).

### c1/GZM^+^ can be divided into GZMK^hi^ and GZMK^+^GZMB^hi^ T_F**K**_ cells

**Figure 6:**
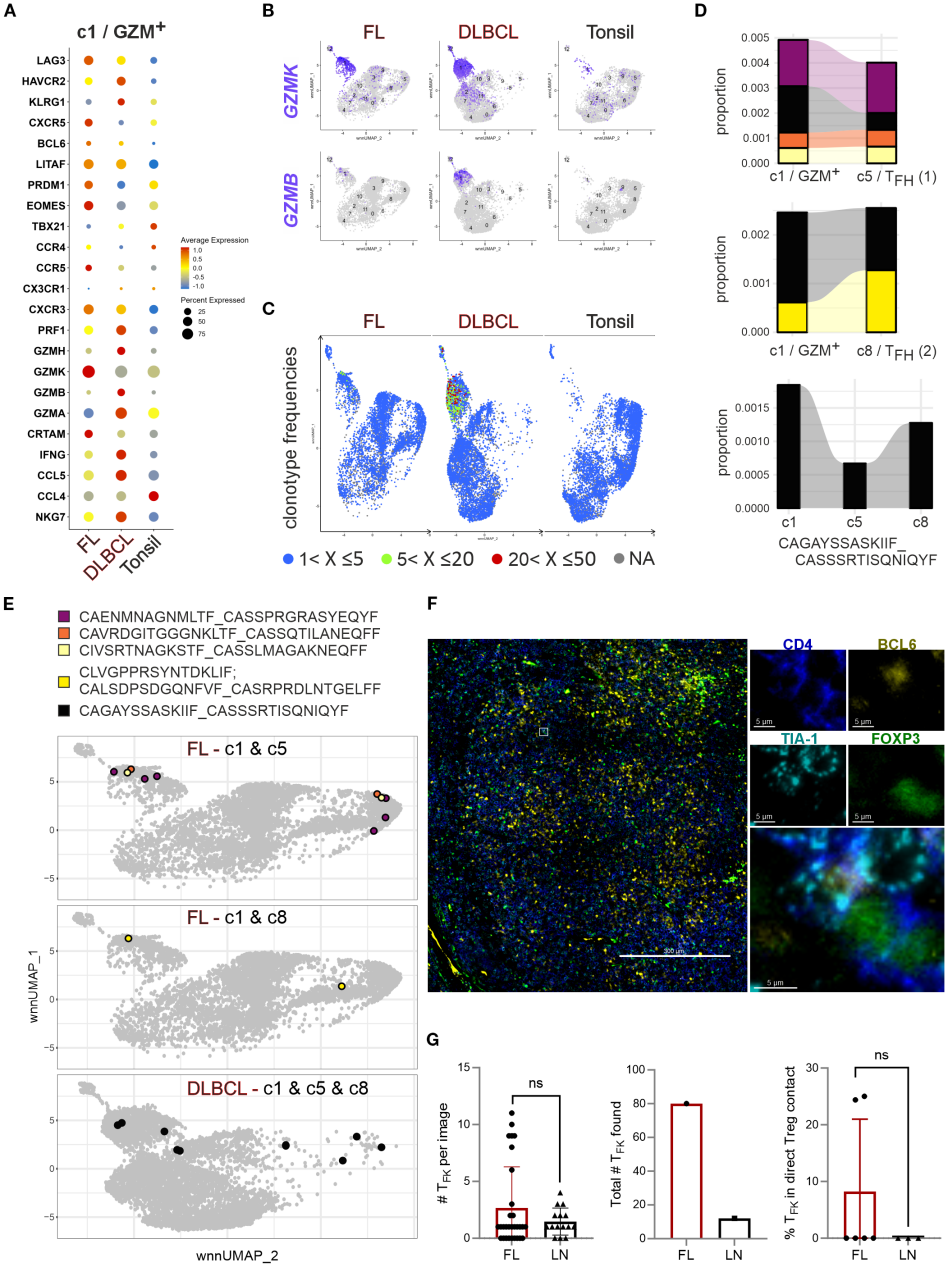
GC-lymphoma-derived T_F**K**_ cells express GZMK +/- GZMB, are oligoclonal, and rarely cluster with Tregs. **(A)** Bubble plot to project T_F_ **_K_** gene markers to c1/GZM^+^ of FL, DLBCL, and tonsils in direct comparison. **(B)** Feature plots of GZMK versus GZMB on simplified wnnUMAPs of FL, DLBCL or tonsillar CD4^+^ T cells. **(C)** TCR clonotype frequencies projected on the wnnUMAPs of FL, DLBCL, and tonsils. The color indicates the rank of a clone in frequency. **(D)** Proportion of enlarged c1-T_F_ **_K_** TCR clones also occurring in c5-T_FH_ (1) and/or c8-T_FH_ (2). The size represents the proportion in the total types. The clonotype sequence shown is shared between c1-T_FK_ and both T_FH_ clusters. **(E)** The color-coded c1 clonotypes shared with c5 and/or c8 are given in sequence and on simplified wnnUMAPs of FL or DLBCL, depending on their occurrence. **(F)** FL with T_F_ **_K_** cells and FOXP3^+^ Tregs: FOXP3 (green), CD4 (blue), BCL6 (yellow), TIA-1 (cyan). Shown is an overlayed overview and a selected T_F_ **_K_** cell in direct contact with a FOXP3^+^ Treg in the four separate channels and as merged image; scale bars 300 µm or 5 µm. **(G)** Number of T_F_ **_K_** cells per image, overall analyzed in this context, and the percentage of T_F_ **_K_** cells in direct contact with Tregs. Mann-Whitney T-test. ns, nonsignificant.

Next, we directly compared c1/GZM^+^ between the different origins (**Figure 6A**). Contrasted to non-malignant tonsils, the level of T_F**K**_ marker gene expression was usually higher in at least one of the lymphomas. Only *CCL4*, *CCR4* and *TBX21* (encoding T-BET) were more pronounced in tonsillar T_F**K**_ cells compared to FL and DLBCL. The follicular phenotype defined by CXCR5 and BCL6 was best observed in FL coinciding with *LAG3*, *LITAF*, *PRDM1*, *EOMES*, *CCR5*, *CXCR3*, and *CRTAM* upregulation. Of note, GZMK was expressed in a high percentage of all cytotoxic CD4^+^ and T_F**K**_ cells, yet the GZMK expression level per cell was clearly chief in FL. c1 cells from DLBCLs exposed their cytotoxic transcriptome with comparably highest expression of *GZMB*, *GZMA*, *GZMH*, *PRF1*, *IFNG*, *CCL5* and *NKG7, HAVRC2 (encoding TIM3) and KLRG1* (**Figure 6A**). The differences were consistent with the appearance of c1 in the UMAPs of FL, DLBCL, and tonsils, defining the dominant phenotype of FL-T_F**K**_ cells as GZMK^hi^ and c1/GZM^+^ as either GZMK^+^ or GZMK^+^GZMB^hi^ enriched in DLBCL (**Figure 6B**).

### T_F**K**_ cells are oligoclonal sharing TCRs with non-cytotoxic T_FH_ cells and Tregs

Our previous study demonstrated an oligoclonal nature of T_F**K**_ cells (15), a finding corroborated by Spasevska et al. through TCR sequencing (32). Clonal expansion was particularly evident in c1/GZM^+^, showing moderate expansion in FL and more prominent in DLBCL (**Figure 6C**). Notably, some of the enriched TCRs in c1 could be found in the T_FH_ clusters (**Figures 6D-E**). A comparison between the two T_FH_ clusters indicated elevated *CXCR4*, *ZNP331*, *TNFAIP3*, and *MAP3K8* transcript levels in c5/T_FH_ (1) (**Supplementary Figure S6**), which is consistent with GC-T_FH_ phenotypes (15). Interestingly, FL-c1/GZM^+^ cells were more likely to share TCRs with GC-T_FH_ (c5) cells than with c8, although c8 was augmented in FL compared to tonsils (**Figure 5C**). One clone defined by a common TCR, appeared in c1, c5, and c8 of DLBCL. Amazingly, the DLBCL-c1-enriched TCR clone found in T_FH_ cells were also present in activated and resting Tregs (**Figure 6E**).

This clonal overlap suggests potential antigen-driven associations, reminiscent of GZMK^+^CD4^+^ T cell behavior in IgG-related diseases (35). However, despite the abundance of GZMK^+^TIA-1^+^ T_F**K**_ and T_FR_/Treg cells in FL, TIA-1^+^ T_F**K**_ cells were rarely detected in direct cell-cell contact with FOXP3^+^ Treg cells (**Figures 6F-G**). Meanwhile, interactions between Tregs and B cells seemed inevitable (**Supplementary Figures S7A-F**). Nevertheless, spatial proximity testing by imaging mass spectrometry found activated Treg/T_FR_ cells to rather interact with other T cells and macrophages than the malignant B cells, while indeed the cytotoxic T_FH_ cells are closer to the malignant B cells (32). These findings collectively indicate that while T_F**K**_ cells are oligoclonal and share TCRs with T_FH_ cells, their activity in lymphoma appears independent of direct Treg-mediated suppression.

### SEB-activated T_F**K**_ cells increase in number, upregulate granzymes and degranulate

**Figure 7:**
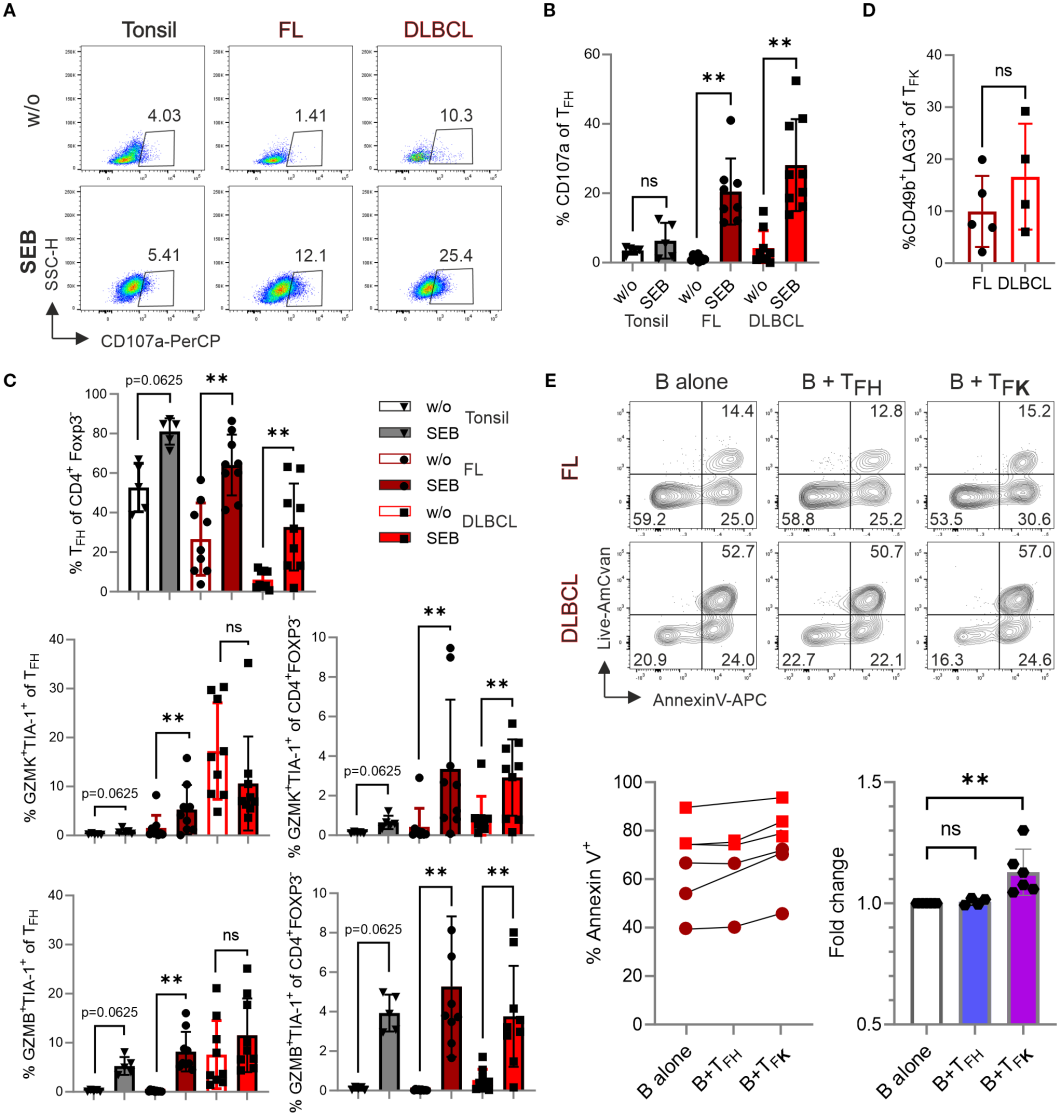
T_F**K**_ cells exhibit cytotoxic activity against B cells. Cells from tonsil (n=5), FL (n=9), and DLBCL (n=9) were stimulated in the presence or absence of 1 μg/mL SEB for 4 days, and different T-cell populations were analyzed by flow cytometry. **(A)** Representative dot plots showing CD107a expression in T_FH_ cells. **(B)** Frequency of CD107a expression in T_FH_ cells. **(C)** Frequency of T_FH_ cells, GZMK^+^TIA-1^+^ and GZMB^+^TIA-1^+^ cells within T_FH_ cells and CD4^+^Foxp3^-^ T cells. **(D)** Frequency of CD49b^+^LAG3^+^ T_R_1 cells within CXCR5^+^PD-1^+^GZMK^+^TIA-1^+^ T_F**K**_ cells. **(E, F)** Representative contour plots **(E)** and frequency **(F)** of Annexin-V^+^ B cells after co-culture with sorted T_FH_ (CXCR5^+^PD-1^+^CD25^–^TIA-1^–^) or T_F**K**_ (CXCR5^+^PD-1^+^CD25^–^TIA-1^+^) cells for 14 hours. Wilcoxon signed-rank test. ns, non-significant, **p < 0.005.

To measure the cytotoxic potential within the CD4^+^CXCR5^+^PD-1^+^ T_FH_ population, we employed the ‘cytokine-independent activation-induced marker’ (AIM) method, which identifies Ag-specific GC-T_FH_ cells (15, 36). Here, SEB activates CD4^+^ T cells in an MHCII-restricted manner. Following four days of SEB stimulation, we observed a significant induction of CD107a surface expression in FL and DLBCL cultures, indicating recent degranulation of cytotoxic granules (37) (**Figures 7A, B**). SEB stimulation not only increased the frequency of T_FH_ cells within CD4^+^FOXP3^–^ Tconv cells, but also significantly expanded GZMK^+^TIA-1^+^ and GZMB^+^TIA-1^+^ T_F**K**_ cells (**Figure 7C**). In FL, the percentage of GZMK^+^TIA-1^+^ and GZMB^+^TIA-1^+^ T_F**K**_ cells within the CD4^+^CXCR5^+^PD-1^+^ T_FH_ population was markedly increased. Interestingly, the dominance of GZMK over GZMB in FL-T_F**K**_ cells was lifted upon SEB stimulation. To ensure a specific T_F**K**_ phenotype, we examined the expression of CD49b and LAG3, which are uniquely co-expressed by CD4^+^ T_R_1 cells—a subset also capable of cytotoxicity (38). Only a minor percentage of T_F**K**_ cells co-expressed CD49b and LAG3 (**Figure 7D**). In sum, GZMK^+^TIA-1^+^ and GZMB^+^TIA-1^+^ T_F**K**_ cells can degranulate and release their cytotoxic cargo upon MHCII-restricted stimulation.

### Isolated T_F**K**_ cells can kill lymphoma-derived B cells *in vitro*

To assess the cytotoxic potential of T_F**K**_ cells, we performed a killing assay using FACS-sorted CD19^+^ B cells (predominantly malignant lymphoma cells) and co-cultured with either CD4^+^PD-1^+^CXCR5^+^TIA-1^–^ T_FH_ or CD4^+^PD-1^+^CXCR5^+^TIA-1^+^ T_F**K**_ FACS-sorted from FL and DLBCL samples (**Supplementary Figures S8A, B**). While isolated GC-B cells (being mostly HLA-DR^+^; **Supplementary Figure S8C**) exhibit poor survival *in vitro*, the presence of T_F**K**_ cells significantly increased the frequency of Annexin V^+^ B cells. This effect was not observed when B cells were co-cultured with T_FH_ cells (**Figure 7E**). These findings suggest that T_F**K**_ cells may exhibit direct cytotoxic activity against lymphoma cells *in vivo*.

## Discussion

We found that GC-derived lymphomas contain a sizable number of PD-1^+^CXCR5^+^ T_F_ **_K_** cells among the T_FH_ cells and among the cytotoxic CD4^+^ T cells, respectively. In FL, they are mostly TIA-1/NKG7^+^GZMK^+^, whereases they are TIA-1^+^GZMK^+^ and/or GZMB^+^ in DLBCL. While B cell non-Hodgkin’s-lymphoma (NHLs) predominantly arise in aged individuals, and age-related T-cell changes—such as cytotoxic CD4^+^ T-cell accumulation, increased circulating T_FH_ (cT_FH_) frequencies, and elevated pre-T_FH_ formation in mice—have been documented (6, 39–41), these alterations do not fully recapitulate the disease-specific T_F**K**_ cell expansion observed in lymphomas. Notably, aged reactive LN samples did not show increased T_F**K**_ cells, supporting that their emergence is lymphoma-driven rather than age-dependent.

DLBCL exhibit a lower frequency of CD4^+^PD-1^+^CXCR5^+^ T cells but a higher proportion of T_F_ **_K_** cells compared to tonsils and FL, with GZMB expression among CD4^+^ Tconv cells being a hallmark of DLBCL. Interestingly, transformation of FL to DLBCL has been associated with a shift from T_FH_ and inflammatory responses to cytotoxic, exhausted phenotypes (42). This progression aligns with shared expression of CXCR5, TCF-1, and BCL6 by T_FH_ and pre-exhausted CD8^+^ T cells, while terminally exhausted CD8^+^ T cells adopt a GZMB^+^CXCR5^–^ profile (43). Collectively, these findings suggest a differentiation trajectory from T_FH_ to GZMK^+^ T_F_ **_K_** to GZMB^+^ T_F_ **_K_** and finally GZMB^+^CXCR5^–^CD4^+^ T-cell phenotypes during lymphoma progression, reflecting the convergence of cytotoxic CD4^+^ and CD8^+^ T cells into exhausted T cells.

The resemblance of GZMB^+^ T_F_ **_K_** to pre-exhausted CD8^+^ T cells does not necessarily imply dysfunction. The bias between either GZMK^+^ or GZMB^+^ cytotoxic CD4^+^ T cells has been observed in bladder cancer (44) and CD4^+^CXCL13^+^ T_FH_-like cells in endometrial cancer (45). Only CD4^+^CXCL13^+^GZMB^+^ T_FH_-like cells harbor neoantigen-specific TCR clonotypes and could be stimulated by neoantigens to further upregulate GZMB, similar to the increase in GZMB expression upon SEB stimulation in FL and DLBCL. Of note, TCR stimulation leads to activation of the *nuclear factor of activated T-cells* (NFAT), which binds to *Gzmb* but not to the other granzyme loci in briefly activated murine CD8^+^ T cells (46). The presence of shared TCR clonotypes among cytotoxic CD4^+^ T cells in both FL and DLBCL implies that recurrent antigen encounter and sustained TCR signaling drive T_F_ **_K_** differentiation, potentially amplified by bystander activation through persistent pro-inflammatory cytokines. Crucially, mature T_F_ **_K_** cells appear to possess functional TCRs, as evidenced by robust GZMB induction in FL-T_F_ **_K_** cells, a notion supported by the proposed MHCII-dependent immune surveillance mediated by cytotoxic T_FH_-like cells in FL (26).

The T_F_ **_K_** cells we found in relatively healthy humans dominantly express the tryptases GZMK and GZMA (15), while in FL they are predominantly GZMK^+^, a phenotype also observed in murine T_FH_ cells provoked by either type-1 infections or immunization (47). There, granzymes mediate distinct cytotoxic mechanisms: GZMB provokes canonical apoptosis by activating the caspase cascade (48), consistent with the ability of isolated and activated T_F_ **_K_** cells to kill lymphoma B cells by inducing apoptosis. In contrast, GZMA leads to activation and release of several proinflammatory cytokines such as IL-1-β as well as processing of gasdermin B, necessary for pyroptosis, altogether inducing an inflammatory form of cytotoxicity. Until recently, the other tryptase, GZMK, was thought to have a mostly overlapping function with GZMA. However, recent studies identify GZMK, but not GZMA, as a thoroughly complement-activating protease (49, 50). Accordingly, CD8^+^GZMK^+^ and CD4^+^GZMK^+^ T cells are enriched at sites of chronic inflammation in the context of autoimmune diseases and tumors likely contributing to chronicity, tumor immune escape, outgrowth and metastasis. Hence, an abundance of GZMA^+^GZMK^+^ cytotoxic CD4^+^ and CD8^+^ T cells was associated with poor survival of B-cell NHL patients (51). On the other hand, in antibody-rich sites like GC-derived lymphomas, immunocomplexes and antibody-dependent cell-mediated cytotoxicity may provide antitumor activity. At least in FL, the high frequency of FL-specific GZMK^+^ T_F_ **_K_** cells correlates with indolence, suggesting a context-dependent protective role. Therapeutically, this aligns with the efficacy of curative antibodies such as anti-CD20, which rely on complement-mediated cytotoxicity (52, 53), highlighting the dual roles of granzyme-mediated pathways in lymphoma progression and treatment. The expression of NKG7/TIA-1 is critically linked to anti-tumor responses, particularly in CD8^+^ T cells (54, 55). In line with GZMK expression and chronic inflammation, NKG7 is upregulated in CD4^+^ T_H_1 and T_R_1 cells (56). In CD8^+^ T cells, NKG7 plays a key role in the exocytosis of cytotoxic molecules. We propose that NKG7 exerts the same function in cytotoxic CD4^+^ T cells including T_F_ **_K_** cells, leading to GZMK-mediated complement activation or GZMB-mediated activation-induced cell death of target – possibly lymphoma – cells.

In IgG-related disease an abundant number of GZMK^+^ cytotoxic T_FH_ cells are clustered with T_FR_ cells, which likely suppress these T_F_ **_K_** cells (35). Similarly, the CD4^+^ T cells being capable of killing autologous human bladder cancer cells, are subject to inhibition by Tregs (44). In FL and DLBCL a high number of Tregs – including T_FR_ cells – are present as well. We detected some but by far not all T_F_ **_K_** cells in direct cell-cell contact with FOXP3^+^ Tregs. It is even possible that the distance to T_FR_/Treg cells warrants IL-2^+^ microdomains supporting BLIMP-1-dependent gain of cytotoxicity and GZMB expression (19). It may be worthwhile to explore whether a tumor-specific Treg approach (32) or specific measures to enhance GC-B lymphoma cell killing by T_F_ **_K_** cells are applicable. One next step will be to clone the TCRs from the expanded T_F_ **_K_** clones and determine their antigen specificity, i.e. whether they are engaged by lymphoma-derived peptides or whether CXCR5^+^ T_F_ **_K_** cells, which naturally home to the atypical follicles, should be equipped with a lymphoma-recognizing CAR.

## Data Availability

The datasets presented in this study can be found in online repositories. The names of the repository/repositories and accession number(s) can be found in the article/**Supplementary Material**.
