# Supplementary material for: Cytotoxic CD4+ T-follicular cells may mediate killing against lymphoma cells
Source: Front Immunol. 2025 Sep 15;16:1657046. doi: 10.3389/fimmu.2025.1657046 (PMC12477175; doi:10.3389/fimmu.2025.1657046)
Supplement: Supplementary file 1 [file DataSheet1.pdf]

Cytotoxic CD4+ T-follicular cells may mediate killing against lymphoma cells

Yin Xiao[1], Sigrun S. Haeusl[1], Gaurav Jethva[1,3], Johannes Weber[1], Andreas Rosenwald[1,2], Friederike Berberich-Siebelt[1,*]

[1]Institute of Pathology, Julius-Maximilians-University Würzburg; Würzburg, Germany.
[2]Comprehensive Cancer Centre Mainfranken, Julius-Maximilians-University of Würzburg; Würzburg, Germany.

[3] present address: Department of Biological and Chemical Engineering, Medical Biotechnology, Aarhus University; Aarhus, Denmark.

* Corresponding author: Prof. Dr. Friederike Berberich-Siebelt, Institute of Pathology, Julius-Maximilians-University of Würzburg, Josef-Schneider-Str. 2, 97080 Würzburg, Germany, Phone: +49 931 31 81208; fax: +49 931 81224; e-mail: path230@mail.uni-wuerzburg.de

- Supplementary data -

Supplementary Table S1

Patients' and donors' baseline characteristics

**Characteristics**	**cohort: FL, n=29**
**Age, years (%)**	
Median (range)	64 (36-81)
Age ≤60 years	9(31)
Age >60 years	20(69)
**Stage (%)**	
FL1-2	21 (72)
FL2-3	8 (28)
**Characteristics**	**cohort: DLBCL, n=9**
**Age, years (%)**	
Median (range)	63 (40-80)
Age ≤60 years	3 (33)
Age >60 years	6(67)
**Characteristics**	**cohort: Tonsil, n=5**
**Age, years (%)**	
Median (range)	5 (2-9)
Age ≤5 years	3 (60)
Age >5 years	2 (40)
**Characteristics**	**cohort: reactive LN, n=6**
**Age, years (%)**	
Median (range)	36 (6-73)
Age ≤40 years	3 (50)
Age >40 years	3 (50)

Supplementary Table S2

Antibodies for flow cytometric staining and FACS sorting

Antigen	Clone	Cat. No	Company
BV510-CD4	OKT4	317444	Biolegend
Percp-CD4	OKT4	317432	Biolegend
FITC-CD4	OKT4	317408	Biolegend
APC-cy7-PD1	NAT105	367416	Biolegend
PE-cy7-PD1	NAT105	367413	Biolegend
Percp-CD19	HIB19	302228	Biolegend
Pacific blue-CXCR5	I252D4	356918	Biolegend
Percp-cy5.5-CD107a	A019D5	351322	Biolegend
PE-cy7-ICOS	C398.4A	313520	Biolegend
PE-TIA-1	2G9A10F5	IM3293	Beckman Coulter
FITC-Foxp3	206D	320106	Biolegend
APC-GZMK	GM26E7	370510	Biolegend
BV510-GZMB	GB11	563388	BD Horizon™
FITC-CD25	BC96	302604	Biolegend
APC-Annexin V		640920	Biolegend

Supplementary Table S3

Antibodies for IF staining

	Antigen	Clone	Host	Cat. No	Company
Primary	CD4	polyclone	Goat	AF-379-NA	R&D
	CD19	6OMP31	Rat	14-0194-82	invitrogen
	BCL6	D4I2V	Rabbit	14895	cell sigaling
	TIA-1 (NKG7)	2G9A10F5	Mouse	IM2550	Beckman Coulter
	FOXP3	PCH101	rat	14-4776-82	invitrogen

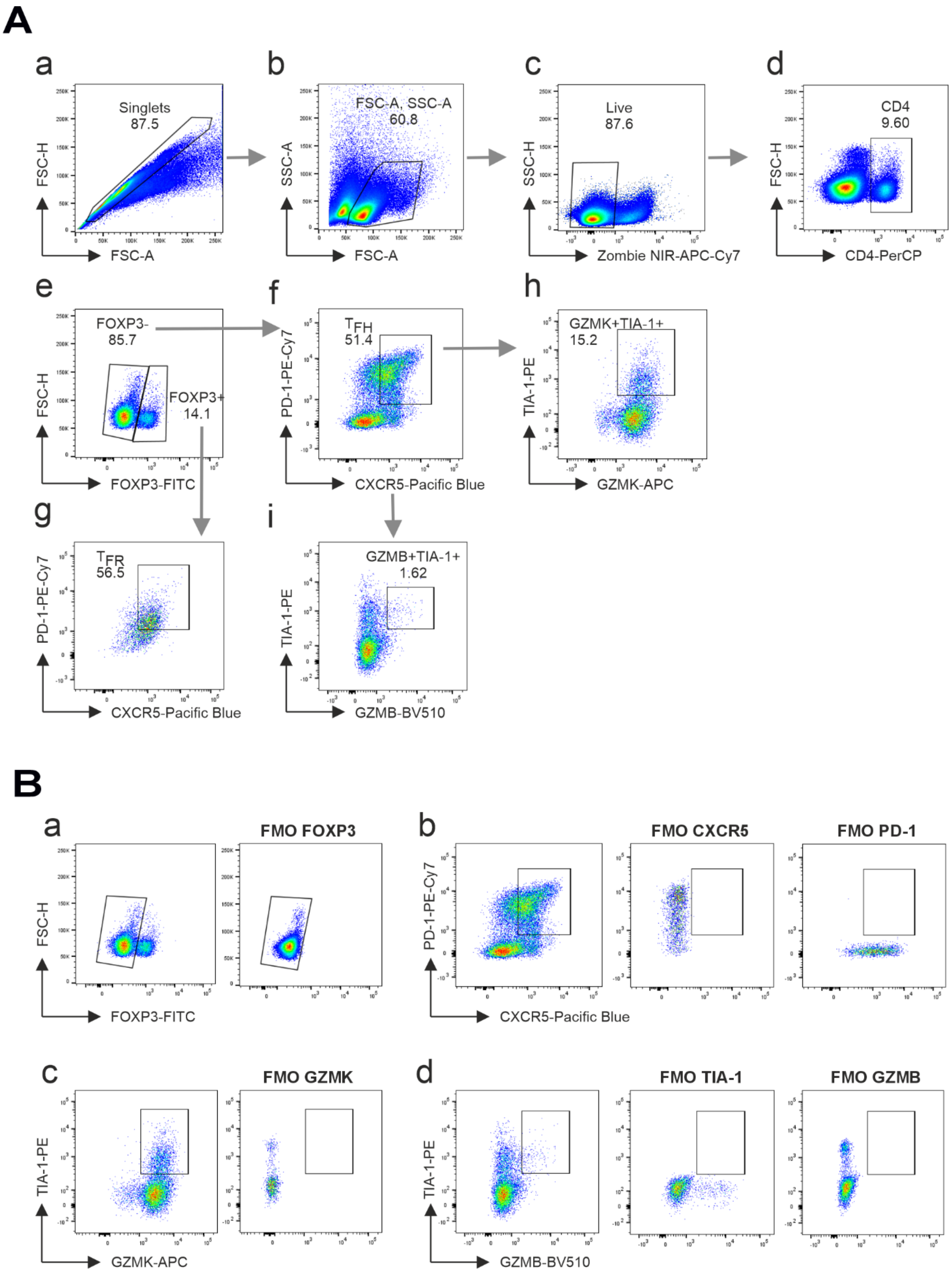

Figure S1

Representative gating strategy. **(A)** Flow cytometry gating strategy used to define cell populations. (a) Singlets. (b) Selection of cells without debris and apoptotic cells. (c) Live cells were gated by

excluding dead cells with Zombie NIR dye (APC-cy7 channel). (d) Selection of $CD4^+$ cells. (e) $CD4^+$ cells were further separated into $FOXP3^-$ (conventional T cells) and $FOXP3^+$ (Treg) subsets. (f) Cells from $Foxp3^-$ were used to analyze $CXCR5^+PD1^+$ T_{FH} cells. (g) Cells from $Foxp3^-$ were used to analyze $CXCR5^+PD1^+$ T_{FR} cells. (h-i) T_{FH} cells were used to analyze $GZMK^+$ TIA-1^+ T_{FK} cells (h) and $GZMB^+$ TIA-1^+ T_{FK} cells (i). **(B)** Fluorescence minus one (FMO) controls were used to establish accurate gating thresholds. (a) Gate used to select $FOXP3^-$ cells (left plot) and respective FMO (right plot). (b) $CXCR5^+$PD-1^+ T_{FH} cell identification (left) with matched FMO controls for CXCR5 and PD-1 (right panels). (c) $GZMK^+$TIA-1^+ T_{FK} cell analysis (left) with GZMK FMO control (right). (d) $GZMB^+$TIA-1^+ T_{FK} cell quantification (left) with FMO controls for both GZMB and TIA-1 (right panels).

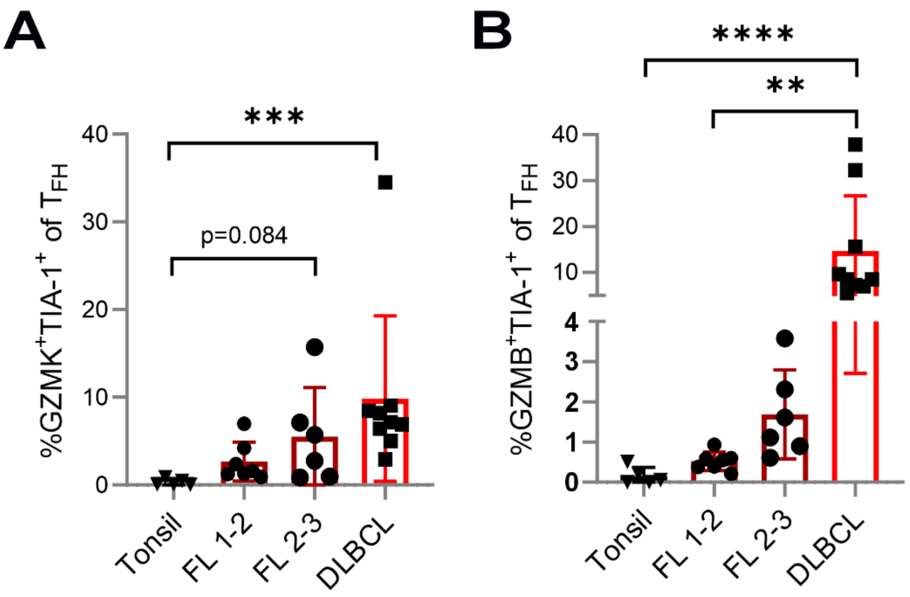

Figure S2

Frequency of T_{FK} cells within the T_{FH} population increases with malignancy. % TIA-1^{+}GZMK^{+} **(A)** and TIA-1^{+}GZMB^{+} **(B)** T_{FK} cells of T_{FH} cells in tonsils, grade 1-2 FL, grade 2-3 FL, and DLBCL. Kruskal-Wallis One-way ANOVA, **$p < 0.005$, *** $p < 0.001$, ****$p < 0.0001$.

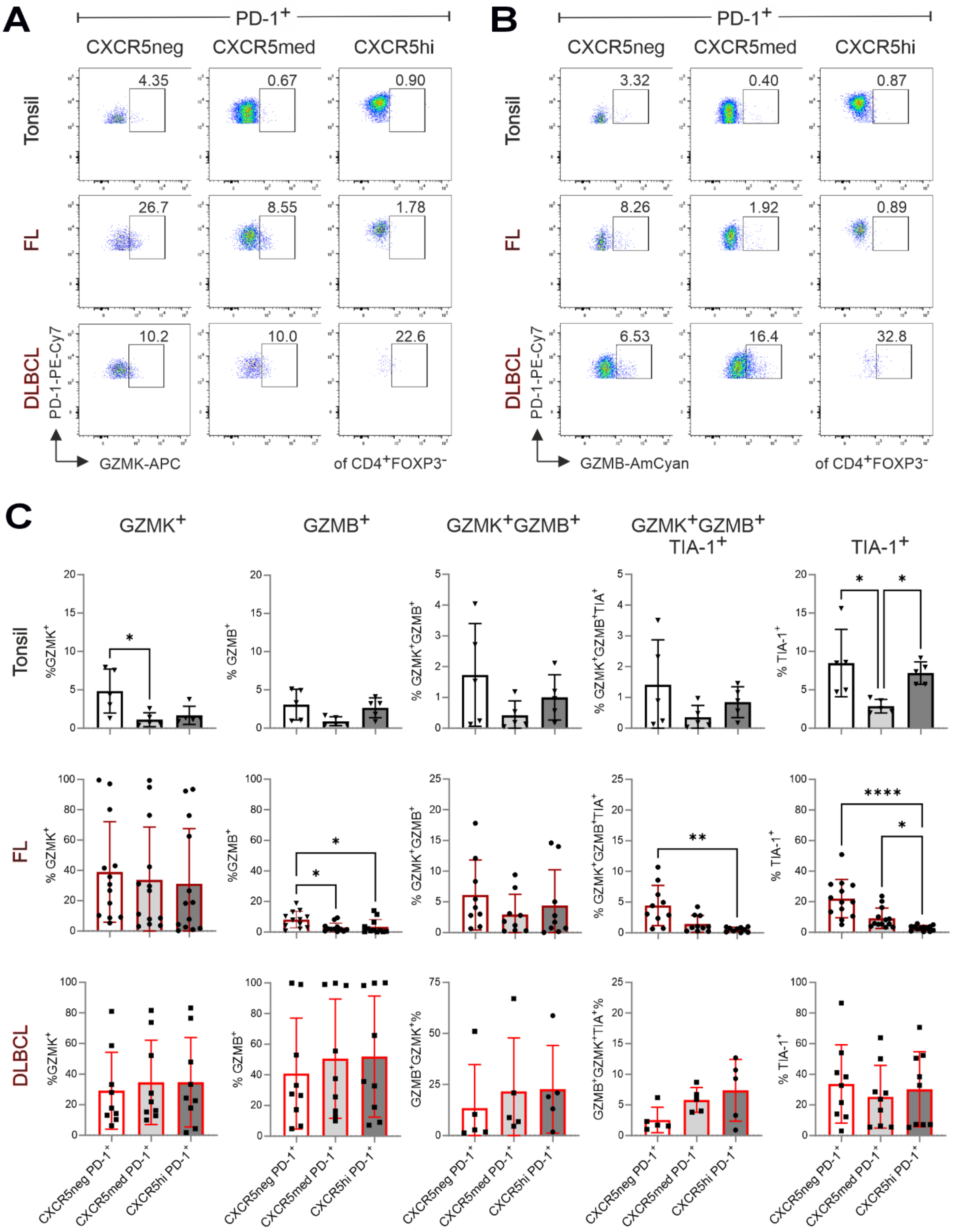

Figure S3

T_{FK} cell frequency decreases with increasing CXCR5 expression in tonsils and FL but remains unchanged in DLBCL – corresponding to Fig. 2. Flow cytometric analysis of T-cell subpopulations in tonsil (n=5), FL (n=13), and DLBCL (n=9). **(A+B)** Representative dot plots of

GZMK (A) and GZMB (B) in $CXCR5^{-}PD\text{-}1^{+}$, $CXCR5^{med}PD\text{-}1^{+}$, and $CXCR5^{hi}PD\text{-}1^{+}$ cells in tonsil, FL, and DLBCL samples. **(C),** Frequency of $GZMK^{+}$, $GZMB^{+}$, $GZMK^{+}GZMB^{+}$, $GZMK^{+}GZMB^{+}TIA\text{-}1^{+}$, and $TIA\text{-}1^{+}$, cells within $CXCR5^{-}PD\text{-}1^{+}$, $CXCR5^{med}PD\text{-}1^{+}$, and $CXCR5^{hi}PD\text{-}1^{+}$ population. Of note, evaluations of single positive $TIA\text{-}1^{+}$, $GZMK^{+}$, and $GZMB^{+}$ represent repetitions of Fig. 2C and are only given again to enable direct comparisons. Kruskal-Wallis One-way ANOVA, * $p < 0.05$, ** $p < 0.005$, **** $p < 0.0001$.

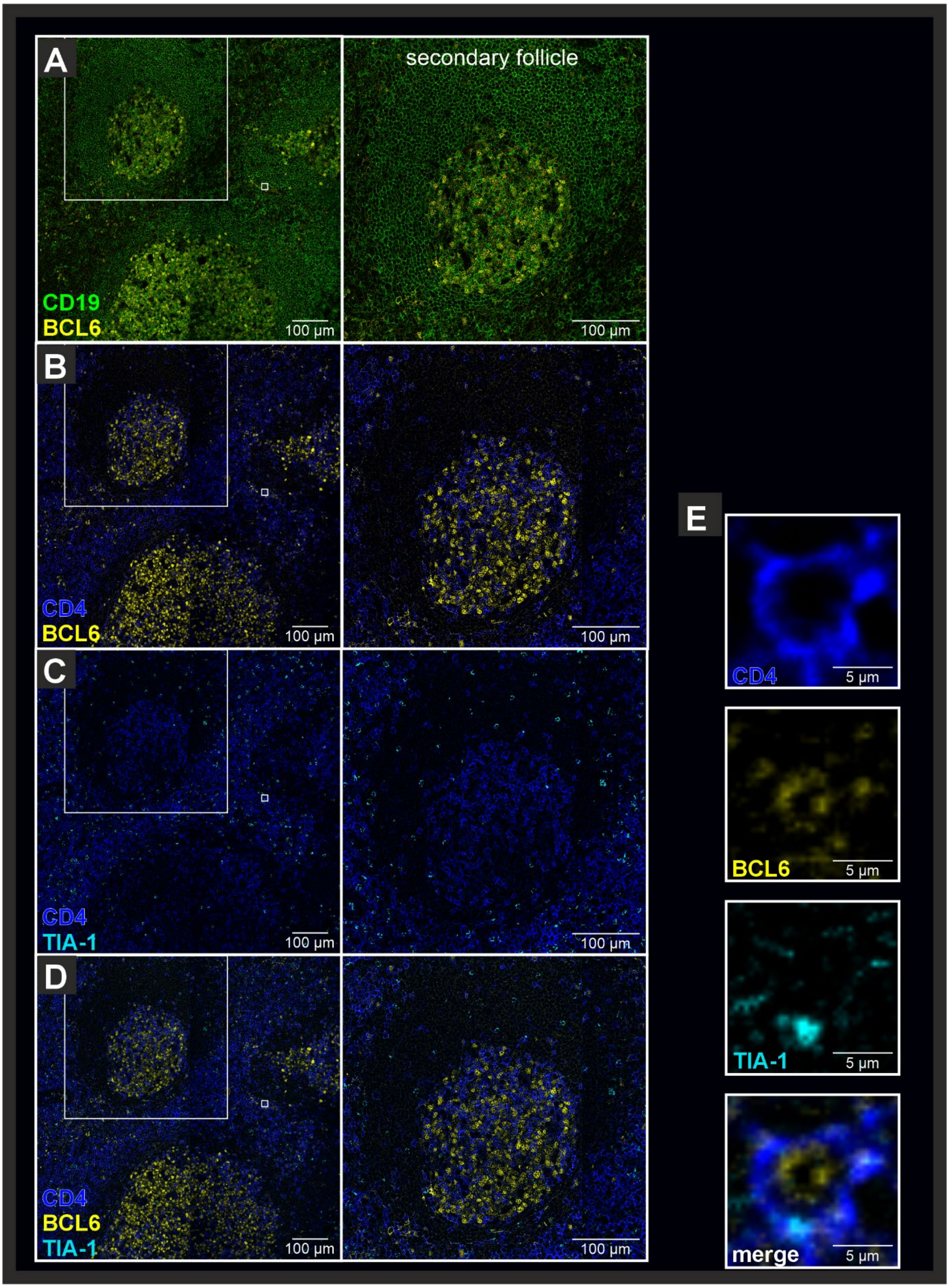

Figure S4

T$_{FK}$ cells can be identified using marker overlap in LN. CD19 (green), CD4 (blue), BCL6 (yellow), TIA-1 (cyan). On the left an overview is depicted, on the right a single secondary follicle is depicted.

(A) Overlay of CD19 and BCL6 staining indicating GC-B cells. **(B)** Overlay of CD4 and BCL6 staining indicating T_{FH} cells. **(C)** Overlay of CD4 and TIA-1 staining indicating cytotoxic $CD4^+$ T cells. **(D)** Overlay of CD4, BCL6 and TIA-1 staining indicating T_{FK} cells. **(E)** Single T_{FK} cell depicted with anti-CD4, -BCL6, and -TIA-1 individually and all antibody staining merged. Figure created with CorelDRAW Graphics Suite.

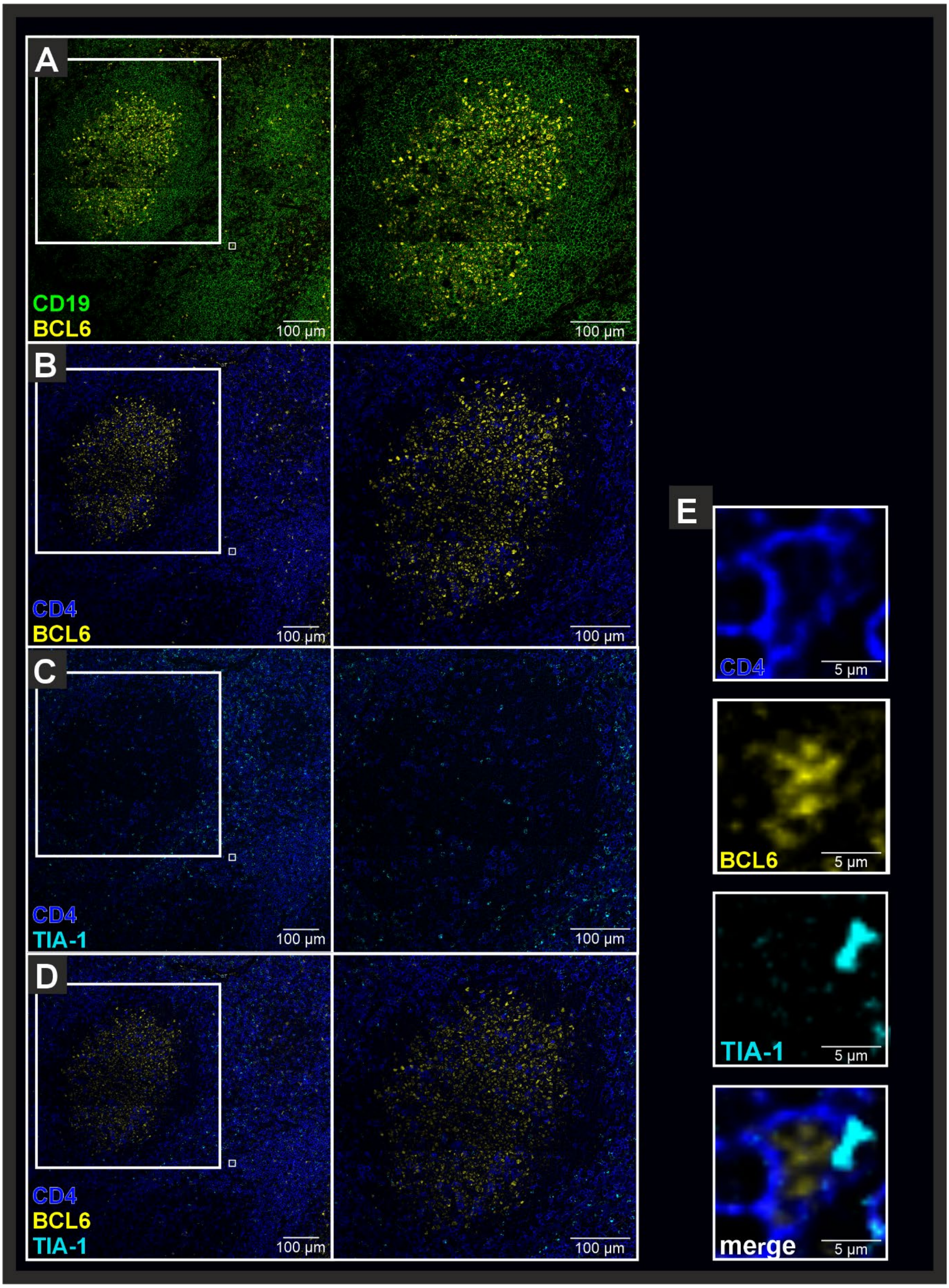

Figure S5

T_{FK} cells can be identified using marker overlaps in FL. CD19 (green), CD4 (blue), BCL6 (yellow), TIA-1 (cyan). On the left an overview is depicted, on the right a single atypical follicle is depicted.

(A) Overlay of CD19 and BCL6 staining indicating GC-B cells. **(B)** Overlay of CD4 and BCL6 staining indicating T_{FH} cells. **(C)** Overlay of CD4 and TIA-1 staining indicating cytotoxic $CD4^+$ T cells. **(D)** Overlay of CD4, BCL6 and TIA-1 staining indicating T_{FK} cells. **(E)** Single T_{FK} cell depicted with anti-CD4, -BCL6, and -TIA-1 individually and all antibody staining merged. Figure created with CorelDRAW Graphics Suite.

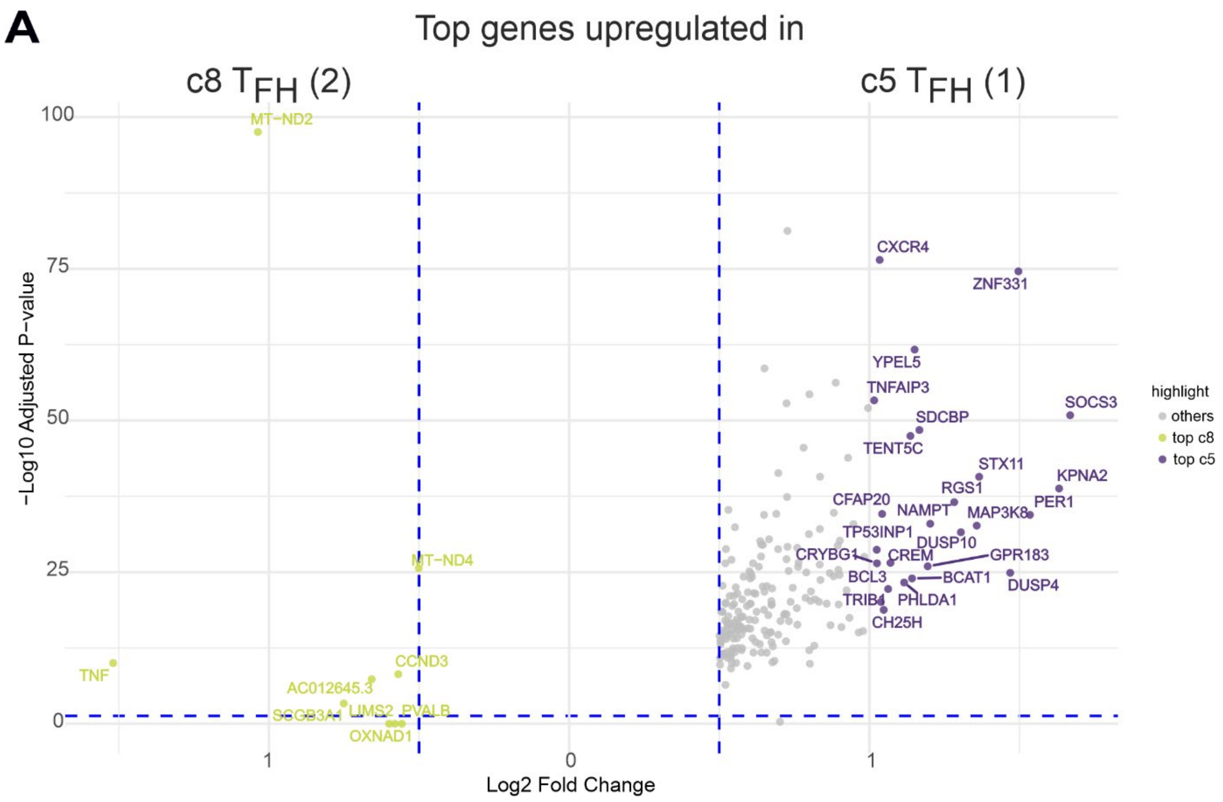

Figure S6

The two T_{FH} cell clusters represent GC-T_{FH} and non-GC-T_{FH} cells. **(A)** Volcano plot for c5 and c8 individually showing differentially upregulated transcripts. On the right, upregulated genes in c5 T_{FH} (purple), on the left, upregulated in c8 T_{FH} (light green).

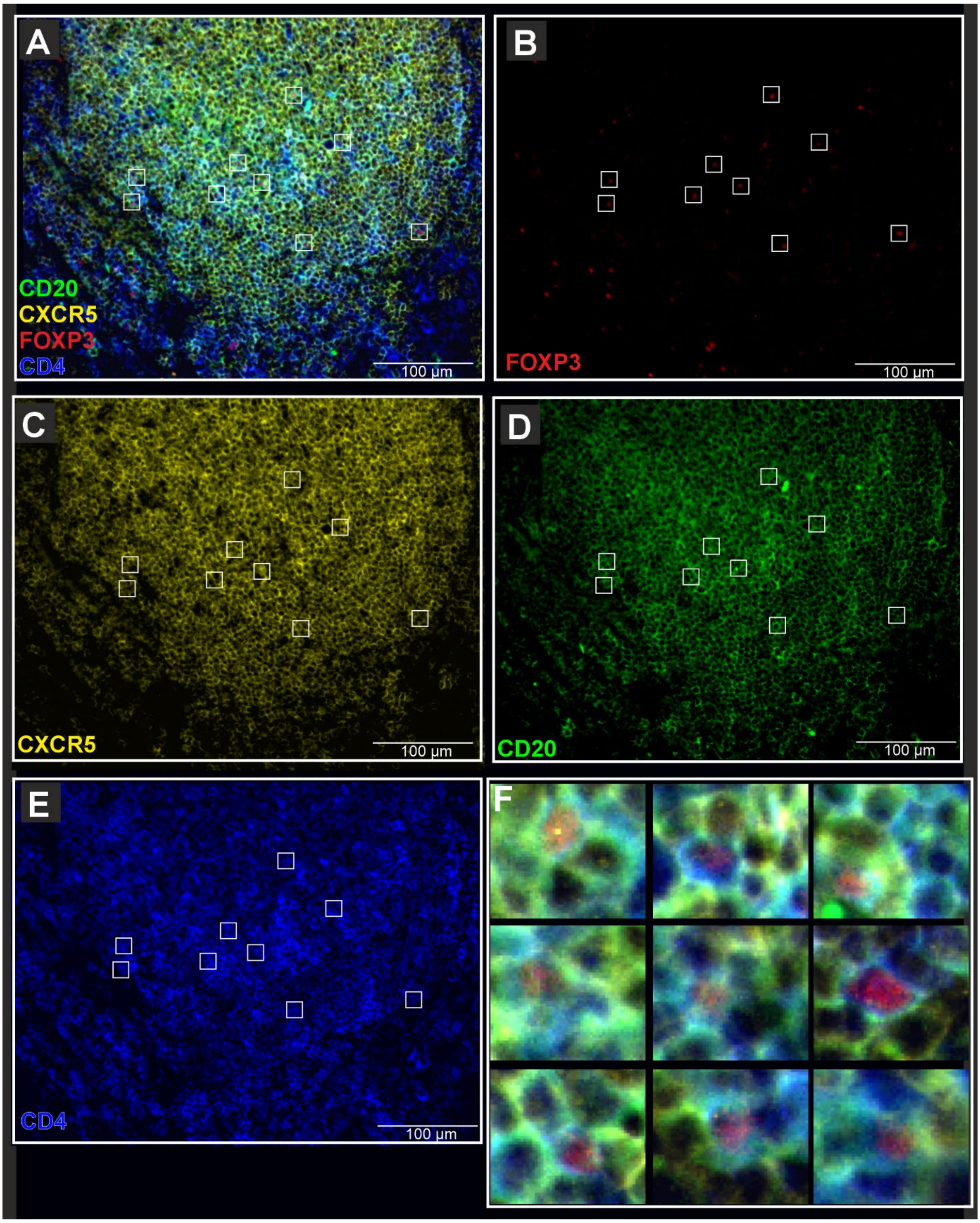

Figure S7

T_{FR} cells are in close contact with B cells in follicular lymphoma (FL) tissue. Immunofluorescence staining of FL tissue sections showing CD20 (green), CD4 (blue), CXCR5 (yellow), and FOXP3 (red). **(A)** Merged overview of all antibody signals. **(B-E)** Individual channels displaying FOXP3 (B), CXCR5 (C), CD20 (D), and CD4 (E), respectively. **(F)** Magnified region highlighting T_{FR} cells ($CD4^+CXCR5^+FOXP3^+$) in direct contact with $CD20^+$ B cells, indicated by a square in panel A.

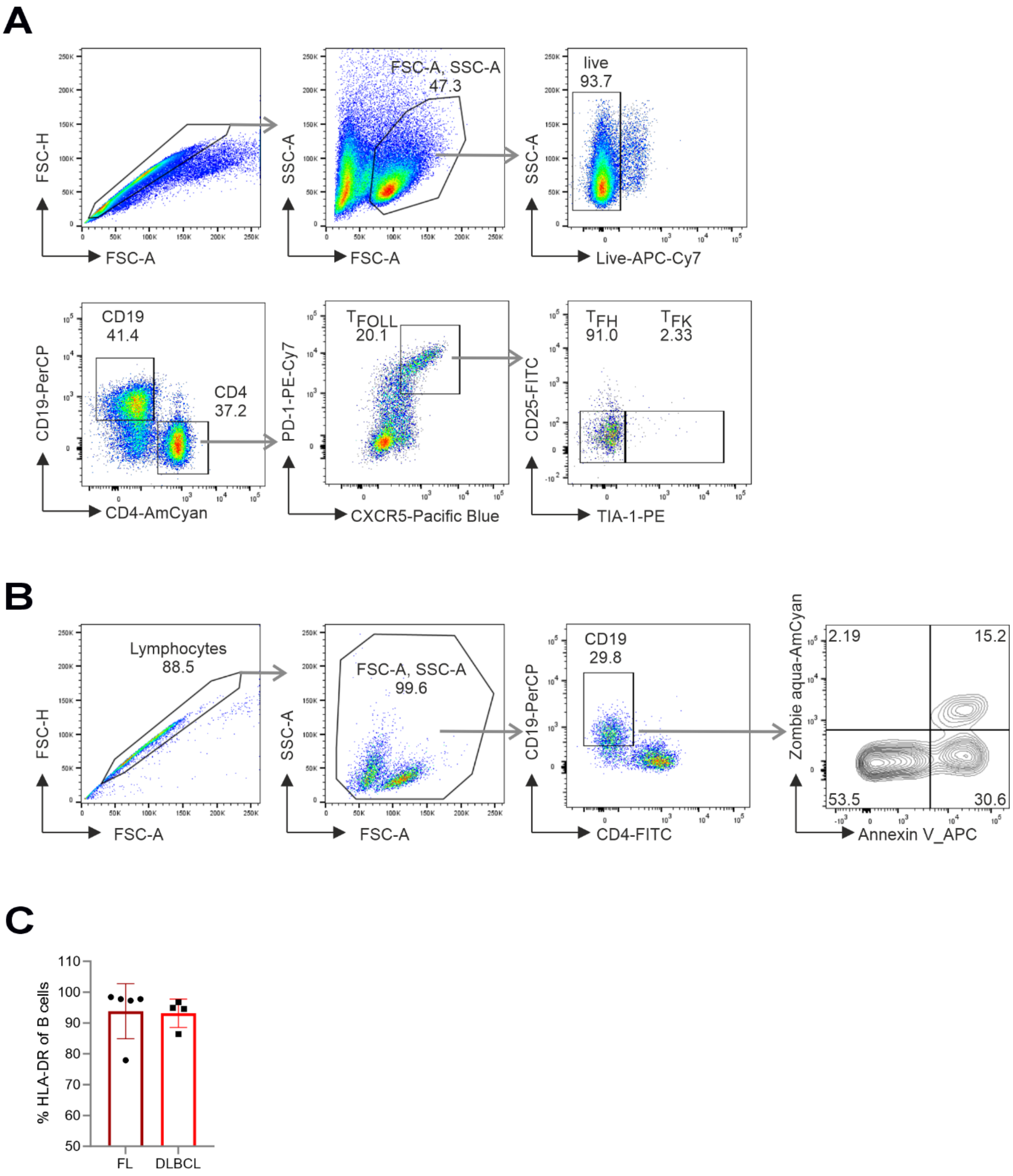

Figure S8

Representative gating for fluorescence-activated cell sorting and the killing assay. **(A)** Gating strategy to sort CD19$^+$ B-FL lymphoma cells and CD4$^+$PD-1$^+$CXCR5$^+$ T-follicular (T_{FOLL}) cells. By exclusion of CD25$^+$ T_{FR} cells, T_{FOLL} cells gave rise to TIA-1$^-$CD25$^-$ T_{FH} cells or TIA-1$^+$CD25$^-$ T_{FK} cells. **(B)** Gating strategy to determine apoptotic CD19$^+$ B-FL lymphoma cells by surface staining of CD19 and live and Annexin V analyses. **(C)** Frequency of HLA-DR$^+$ FL and DLBCL cells.
